# Three tyrosine kinase inhibitors cause cardiotoxicity by inducing endoplasmic reticulum stress and inflammation in cardiomyocytes

**DOI:** 10.1186/s12916-023-02838-2

**Published:** 2023-04-17

**Authors:** Huan Wang, Yiming Wang, Jiongyuan Li, Ziyi He, Sarah A. Boswell, Mirra Chung, Fuping You, Sen Han

**Affiliations:** 1 Institute of Systems Biomedicine, School of Basic Medical Sciences, Peking University Health Science Center, Beijing, 100191 China; 2 Laboratory of Systems Pharmacology, Department of Systems Biology, Harvard Medical School, Boston, MA 02115 USA; 3 Key Laboratory of Carcinogenesis and Translational Research (Ministry of Education), Peking University Cancer Hospital & Institute, Beijing, 100142 China

**Keywords:** Cardiotoxicity, Tyrosine kinase inhibitor, Transcriptomics, Endoplasmic reticulum stress, Inflammation

## Abstract

**Background:**

Tyrosine kinase inhibitors (TKIs) are anti-cancer therapeutics often prescribed for long-term treatment. Many of these treatments cause cardiotoxicity with limited cure. We aim to clarify molecular mechanisms of TKI-induced cardiotoxicity so as to find potential targets for treating the adverse cardiac complications.

**Methods:**

Eight TKIs with different levels of cardiotoxicity reported are selected. Phenotypic and transcriptomic responses of human cardiomyocytes to TKIs at varying doses and times are profiled and analyzed. Stress responses and signaling pathways that modulate cardiotoxicity induced by three TKIs are validated in cardiomyocytes and rat hearts.

**Results:**

Toxicity rank of the eight TKIs determined by measuring their effects on cell viability, contractility, and respiration is largely consistent with that derived from database or literature, indicating that human cardiomyocytes are a good cellular model for studying cardiotoxicity. When transcriptomes are measured for selected TKI treatments with different levels of toxicity in human cardiomyocytes, the data are classified into 7 clusters with mainly single-drug clusters. Drug-specific effects on the transcriptome dominate over dose-, time- or toxicity-dependent effects. Two clusters with three TKIs (afatinib, ponatinib, and sorafenib) have the top enriched pathway as the endoplasmic reticulum stress (ERS). All three TKIs induce ERS in rat primary cardiomyocytes and ponatinib activates the IRE1α-XBP1s axis downstream of ERS in the hearts of rats underwent a 7-day course of drug treatment. To look for potential triggers of ERS, we find that the three TKIs induce transient reactive oxygen species followed by lipid peroxidation. Inhibiting either PERK or IRE1α downstream of ERS blocks TKI-induced cardiac damages, represented by the induction of cardiac fetal and pro-inflammatory genes without causing more cell death.

**Conclusions:**

Our data contain rich information about phenotypic and transcriptional responses of human cardiomyocytes to eight TKIs, uncovering potential molecular mechanisms in modulating cardiotoxicity. ER stress is activated by multiple TKIs and leads to cardiotoxicity through promoting expression of pro-inflammatory factors and cardiac fetal genes. ER stress-induced inflammation is a promising therapeutic target to mitigate ponatinib- and sorafenib-induced cardiotoxicity.

**Graphical Abstract:**

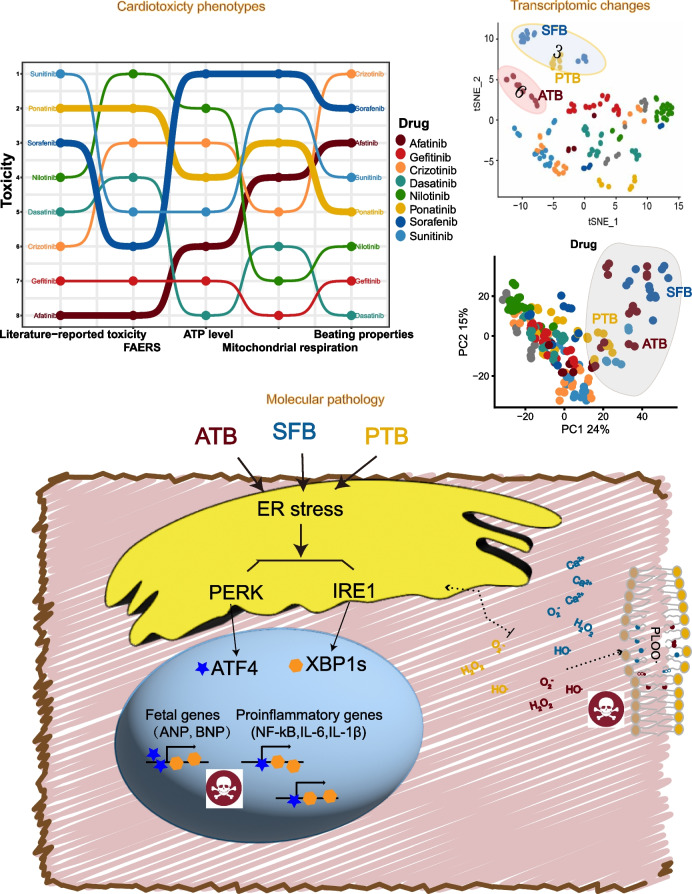

**Supplementary Information:**

The online version contains supplementary material available at 10.1186/s12916-023-02838-2.

## Background

Tyrosine kinase inhibitors (TKIs) are widely prescribed in the clinic to treat cancer from blood malignancy to advanced solid tumors. As drugs that specifically target overexpressed or hyperactivated signaling downstream of receptor tyrosine kinases, TKIs are viewed as much safer than traditional chemotherapy, such as doxorubicin. But TKIs are often used for many months without a dosage cap, some of them still cause serious cardiac adverse events. For example, sunitinib is associated with 4.1% of congestive heart failure in a meta-analysis of 6935 patients [1] and sorafenib causes 2.7–3% of myocardial ischemia in clinical trials [2, 3]. Both TKIs cause hypertension in up to 47% of patients, which is probably due to inhibition of the VEGF signaling [4]. In another study, 14% of patients with nilotinib treatment have one or more cardiovascular events, including peripheral artery disease (10%) and myocardial infarction (4%) [5]. Ponatinib is associated with a significant incidence of arterial occlusion events (26%) [6] and other adverse cardiac events, including arrhythmia, hypertension, and myocardial infarction [7]. Those TKIs cause a wide array of cardiovascular toxicities in noticeable fractions of patients; yet clinical management is limited. Drug holiday, dose reduction, or standard anti-heart failure therapies are used depending on the type and severity of cardiotoxicity. To improve life quality and clinical treatment of patients with drug-induced cardiotoxicity, we need to understand molecular mechanisms of the TKI-induced cardiotoxicity.

TKI-induced cardiotoxicity can be classified into two categories: “on-target” and “off-target” effects. In these categories, cardiac cells develop adaptive and maladaptive responses to the pharmacological effects of TKIs. The physiologically inherited responses of the hearts, such as the hypertrophic response, the fetal gene program, the unfolded protein response, and the antioxidant response, are often induced by exogenous chemical insults and may regulate cardiotoxicity adaptively over time. TKI-induced cardiotoxicity is determined by both the pharmacological inhibition of putative targets or off-targets and the stress responses of cardiac cells. By profiling 4 TKIs at three different doses and four time points, we found that transcriptomic changes facilitate identification of cellular stress responses [8]. Cardiac cells share a similar adaptive or drug-resistant pathway of aerobic glycolysis with cancer cells in response to sorafenib [8]. A later study expanded the transcriptome profiling to 26 TKIs at a fixed dose and time and found that cardiac cell-based transcriptomic changes in combination with structure–activity relation of TKIs are predictive of drug-induced cardiotoxicity risks [9]. Therefore, the high-throughput transcriptomics of human cardiomyocytes can improve our understanding on mechanisms of cardiotoxicity caused by TKIs.

One of the most conserved stress responses is the endoplasmic reticulum (ER) stress, which is active in nearly all cell types of human tissues. Changes in the oxidative, calcium, lipid modification, and unfolded protein levels in the ER can trigger this response, which activates three effectors, inositol-requiring enzyme 1α (IRE1α), protein kinase R-like endoplasmic reticulum kinase (PERK) and ATF6 [10]. IRE1α activates the expression of ER chaperons and ER-associated degradation (ERAD) components to reduce ER stress [11]. However, it may engage TRAF2 to cause apoptosis [12] or NF-κB to induce inflammation [13]. PERK reduces folding load in ER through phosphorylating translation initiation factor 2α (eIF2α), which inhibits mRNA translation [14]; however, it may induce apoptosis through ATF4 and CHOP (official gene name: *DDIT3*) upregulation [15] or activate IL6 to promote inflammation [16]. The primary role of ATF6 in the heart is to preserve proteostasis [17]. Therefore, the three effectors downstream of ER stress have both positive and negative effects on cell fate, dependent on the selective pathways activated, or the duration of ER stress. Genetic modulation of the three effectors in mice hearts show a protective role of ER stress in response to pressure overload or ischemic diseases [18–21]. However, if CHOP downstream of the PERK-ATF4 signaling is activated, this causes aggravated cardiac damage induced by ER stress [22]. Since cardiac adverse effects induced by TKIs share similar phenotypes as pressure overload or ischemia, ER stress may be activated in TKI-induced cardiotoxicity. Indeed, imatinib-induced cardiotoxicity is associated with PERK and IRE1α activation and nilotinib activates ER stress and cell death in rat cardiac H9C2 cells [23, 24]. However, which downstream selected pathway(s) or how the activation duration of ER stress regulates cardiotoxicity remains unknown.

To systematically study and compare cardiotoxicity mechanisms of TKIs, we profiled phenotype and transcriptome of human cardiomyocytes in a high throughput manner with over 100 treatments of eight TKIs. The transcriptome analysis not only helps us understand the specific biological processes regulated by the TKIs in cardiomyocytes but also enables comparison among TKIs with similar or different targets. ER stress is one of the highly enriched pathways regulated by three TKIs and how ER stress induced by TKIs promotes cardiotoxicity through inflammation and fetal gene re-expression, rather than cell death, is explored in this study.

## Methods

### Human induced pluripotent stem cell-derived cardiomyocytes (hiPSC-CM) culture and drug treatment

Cor.4U hiPSC-CMs derived from female human cells were ordered from Ncardia (https://www.ncardia.com) and maintained in the manufacturer’s media. Cells were grown in growth media (Cor.4U Complete Culture Medium which contains 10% FBS and other essential nutrients, Catalog number Ax-M-HC250 from Ncardia) and treated in minimal media (BMCC Serum-Free Culture Medium, Catalog Number Ax-MBMCC250 from Ncardia, supplemented with 1% fetal bovine serum from Thermo Fisher Scientific) in a humidified incubator with 5% CO_2_ and 37 °C. Cryo-preserved hiPSC-CMs were thawed into 75-cm^2^ flasks, cultured in growth media for 3 days, and reseeded into multi-well plates. Then, hiPSC-CMs were cultured in minimal media for 1.5 days prior to drug treatment and maintained in minimal media during drug treatment with media exchanged every other day. Drug stocks were usually prepared in DMSO at 10 mM and stored at − 20 °C until use.

### Neonatal rat cardiac myocytes (NRCM) isolation, culture, and treatment

Dissection of the hearts was performed on 1–2 days old Sprague–Dawley rats (Beijing Vital River Laboratory Animal Technology) and the procedure did not involve the use of anesthetics. Rats were euthanized by decapitation and thoracic cavity was opened with scissors and hearts were popped out of the opening gently with fingers. Hearts were clipped and put in HBSS immediately; 20–40 hearts were collected into one sterile vial containing a magnetic stir bar and 1 mL digestive media made of 0.1% trypsin (Macklin) and 0.05% collagenase (Gibco) in HBSS. Hearts were minced until they were uniform in size. Digestive media was added to 10 mL for 40 hearts and stirred gently for 6 min. Supernatant was discarded and the digestion was repeated once; 10 mL digestive media was added and stirred gently for 10 min. The supernatant with cells were collected and the digestion and collection were repeated 4 times. Cells were centrifuged at 1200 rpm for 5 min, resuspended in media (Dulbecco’s modified Eagleʼs medium (Gibco)) with 10% fetal bovine serum (FBS; VISTECH), 1% penicillin–streptomycin, and 1% 0.1 M 5-bromo-2-deoxyuridine solution (Shanghai Yuanye) and filtered through a 40-μm cell strainer. Cells were seeded on a 100-mm plastic dish and cultured in a 5% CO_2_ incubator at 37 °C for 2 h to allow non-myocytes to adhere to the plate. The dish was swirled and media gently pipetted up and down to detach lightly attached cardiomyocytes. The collected myocytes were seeded in the same media described before; 48 h after plating, medium was exchanged to fresh DMEM (Gibco) with or without 2% FBS. All drug treatments were performed after this media change.

### 3’digital gene expression with unique molecular identifiers (3’DGE-UMI) RNAseq and data analysis

Cor.4U hiPSC-CMs were cultured in 96-well plates, precultured as described in “human induced pluripotent stem cell-derived cardiomyocytes (hiPSC-CM) culture and drug treatment” and treated with drugs as shown in Fig. 2A. Total RNA was isolated using MagMAX™-96 total RNA isolation kit. RNA was transferred into 384-well plates and the subsequent library preparation was done according to this publication [25] and using an automated liquid dispensing system. RNA was reverse transcribed using the Maxima H Minus Reverse Transcriptase (Thermo Fisher) and barcoded primers containing poly(T) sequence about 24 bases long, a random 10-nucleotide sequence as unique molecular identifiers, a random 6-nucleotide sequence as well barcodes, and a sequence complimentary to pre-amplification primers. The other end of cDNA sequences was filled with a sequence complementary to pre-amplication primers through a template switching oligo and the same reverse transcriptase. The cDNAs of different drug treatments were pooled and amplified by PCR.

The cDNA library was sequenced twice, once in a Nextseq 500 (illumina) in the Bauer Core of Harvard University with 17 cycles on Read 1 and 60 cycles on Read 2 and another time in a Novaseq 6000 (illumina) using BerryGenomics with 150 cycles on both Reads. Even though the second sequencing yielded double the amounts of counts per sample, the t-distributed Stochastic Neighbor Embedding (tSNE) clustering of sequencing results was very similar to the previous data and we used the later data for analysis. Data were analyzed using R version 4.1.0 and Seurat_4.0.3.

### Statistical analyses

All data are presented as mean ± SEM. Data were analyzed using a two-tailed Student’s *t*-test (for two-grouped comparisons) or ANOVA (for multiple-group comparisons). Significance was assigned at *p* < 0.05, *p < 0.05, **p < 0.01, ***p < 0.001, n.s. not significant. In all figures, *n* referred to the sample size which was selected based on previous studies. Unless otherwise indicated, the results were based on a minimum of three independent experiments to ensure reproducibility. Statistical analyses were performed using GraphPad Prism 9.0 software (GraphPad Software Inc., La Jolla, CA, USA). Adobe illustrator 27.1.1 was used to create artwork (Adobe Inc., USA).

Detailed methods were presented in the supporting materials.

## Results

### A thorough analysis of cardiotoxicity of eight TKIs on cardiomyocytes and in patient data

At first, an in vitro human cardiomyocyte-based cell culture system was established to classify TKIs with different levels of cardiotoxicity. We selected TKIs with low cardiotoxicity (afatinib, gefitinib), medium-levels of cardiotoxicity (crizotinib, dasatinib, nilotinib), and high-levels of cardiotoxicity (sorafenib, sunitinib and ponatinib) based on literature reports (Table S1) [2, 4, 26–36]. Afatinib and gefitinib were reported without or with low cardiotoxicity. Crizotinib, dasatinib, and nilotinib induced cardiotoxicity at medium-level, including bradycardia, cardiac ischemia, and periphery vascular occlusion. Ponatinib, sorafenib, and sunitinib can target VEGFR2 and PDGFRs, so that cause high-levels of cardiotoxicity, such as hypertension, heart failure, myocardial infarction, and cardiac arrhythmias (Table S1). As it remains debatable, TKIs cause cardiotoxicity mainly through on-target or off-target effects and limited studies compared the cardiotoxic mechanisms of TKIs with similar targets, we chose 2–3 drugs that target EGFR (afatinib, gefitinib), Bcr-Abl (dasatinib, nilotinib), or VEGFR/PDGFR (ponatinib, sorafenib and sunitinib) so as to address these questions. The 8 TKIs selected are also widely used to treat different cancer types, from leukemia to solid tumors. Two previous studies have evaluated over 20 FDA-approved TKIs at a single dose and treatment duration on hiPSC-CMs or primary human cardiac cells [9, 37]; we think that we could gain different insights from the previous studies by studying more doses and treatment durations which mimic more variables associated with clinic usage of these drugs in order to find critical cardiotoxic mechanisms.

**Fig. 1:**
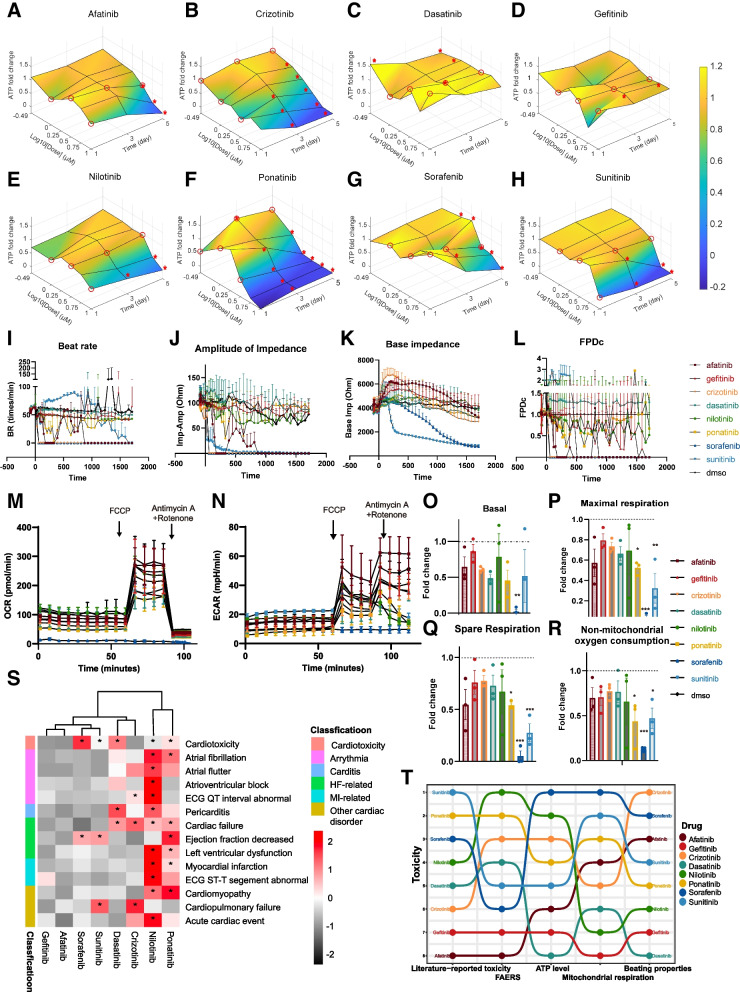
Toxicity of eight TKIs measured by cellular assays, FAERS analysis, and literature review.** A**–**H** Cor.4U hiPSC-CMs were treated with TKIs at doses from 0.32 to 10 µM and duration from 1 to 5 days. Fold changes in ATP were calculated relative to vehicle controls at the same time point and shown as a surface plot. Treatments selected for subsequent RNAseq profiling were enclosed in red circles. Toxicity that was significantly different from controls was labeled with red asterisks. **I**–**L** Base impedance and extracellular field potential were measured by CardioExcyte 96 microelectrode array in HELP hiPSC-CMs treated with fixed doses of TKIs (afatinib 10 µM, gefitinib 10 µM, crizotinib 3.16 µM, dasatinib 10 µM, nilotinib 10 µM, ponatinib 3.16 µM, sorafenib 10 µM, sunitinib 10 µM) for 24 h. Beat rate (**I**), amplitude of impedance (**J**), base impedance (**K**), and corrected field potential duration (FPDc, **L**) were calculated for different treatments. Data were presented as mean ± SD of three replicated wells. **M**–**N** Mitochondrial oxygen consumption and extracellular acidification were measured in rat cardiomyocytes treated with fixed doses of TKIs (same as in **I**) for 24 h. A representative experiment from three independent repeats was shown. **O**–**R** Basal, maximal, spare, and non-mitochondrial oxygen consumption were derived from the seahorse experiment from **M**. Data were presented as mean ± SEM of three independent experiments with 5–6 replicated wells each. *p < 0.05, **p < 0.01, ***p < 0.001 versus the DMSO vehicle control group. **S** Heatmap of reporting odds ratios (RORs) calculated based on the event numbers of cardiotoxicity-related medical terms mined from the FDA adverse events reporting system (FAERS). **T** Toxicity rankings of eight TKIs based on literature, FAERS, ATP level, mitochondrial respiration and beating properties. Drug with the highest toxicity is on the top

To evaluate the toxic effects of TKIs on cardiomyocytes, we assessed the ATP level, contraction, and respiration in response to different doses and treatment durations. ATP levels were measured over a dose range from 0.32 to 10 µM (spanning therapeutic relevant doses of the drugs) and over a time range from day 1 to day 5. Most drugs (afatinib, crizotinib, nilotinib, ponatinib, and sunitinib) caused dose-dependent and time-dependent reduction in ATP levels in hiPSC-CMs, whereas dasatinib and gefitinib did not inhibit ATP at any dose or time point tested (Fig. 1A–F, H). Sorafenib caused slight increase in the ATP level at day 1, but decrease at day 5 (Fig. 1G). We fitted a four-parameter log-logistic model to each dose response curve to calculate EC50. For drugs that shown inhibitory effects on ATP, EC50s were from 1 to 5 μM at day 5 (Fig. S1A-H) and decreased from day 1 to day 5, indicating an increase in toxicity over time (Fig. S1I). EC50s of sorafenib and nilotinib were lower than or similar to their maximal plasma concentrations (*C*_max_), while EC50s were higher than *C*_max_ for the other drugs (Fig. S1A-H). We also used microelectrode array to measure base impedance and extracellular field potential (EFP) of hiPSC-CMs in response to these TKIs. Sorafenib (10 µM) and crizotinib (3.16 µM) stopped beating and contraction of hiPSC-CMs after 0.5 h of treatment (Fig. 1I, J). Afatinib (10 µM) and ponatinib (3.16 µM) reduced beat rate and corrected field potential duration (Fig. 1I, L), while nilotinib, gefitinib, and dasatinib had minimal effects on these parameters (Fig. 1I–L). Sunitinib (10 µM) increased beat rate initially and reduced the base impedance very acutely, indicating cell dissociation or death induced by the drug (Fig. 1I, K).

Since many TKIs affected the ATP level in hiPSC-CMs, we evaluated the effects of these TKIs on mitochondrion, the main organelle for ATP generation, through both the seahorse assay and measuring mitochondrial membrane potential. After 24 h of treatment, ponatinib, sorafenib, and sunitinib inhibited maximal, spare, and non-mitochondrial oxygen consumption significantly (from *p* < 0.05 to *p* < 0.001, Fig. 1M, P, Q, R). Many TKIs showed the trend of inhibiting basal oxygen consumption rate (OCR), such as afatinib, crizotinib, dasatinib, ponatinib, and sunitinib, but only sorafenib had a significant inhibition (*p* < 0.01, Fig. 1O). In the acute treatment, most TKIs did not inhibit basal OCR, indicating that these TKIs are not acutely toxic to mitochondria (Fig. S2A). Based on the quantification of mitochondrial membrane potentials, only sorafenib decreased the mitochondrial membrane potential significantly after 24 h of treatment; nilotinib, ponatinib, and sunitinib showed the trend of decreasing mitochondrial membrane potentials (Fig. S3). The inhibitory effects of TKIs on mitochondria cannot fully explain the changes in the ATP level. Extracellular acidification rate, which correlates with cellular glycolysis rate, was not significantly affected by these TKIs (Figs. 1N and S2B). To further quantify the cardiotoxicity events induced by these TKIs in patients, we analyzed the USA federal drug administration adverse event reporting systems (FAERS) and showed that nilotinib and ponatinib caused the most cardiotoxicity with significant reporting odds ratios (RORs), followed by crizotinib and dasatinib (Fig. 1S). The overall rankings of toxicity of these TKIs based on the literature review, the FAERS, the ATP level, mitochondrial respiration, and beating properties were similar, but the toxicity of afatinib was rated higher in cellular assays than in FAERS or literature, and the toxicity of dasatinib was rated lower in cellular assays than in FAERS or literature (Fig. 1T).

### TKI-induced transcriptome changes are grouped into 7 clusters, with two enriched in ER stress

**Fig. 2:**
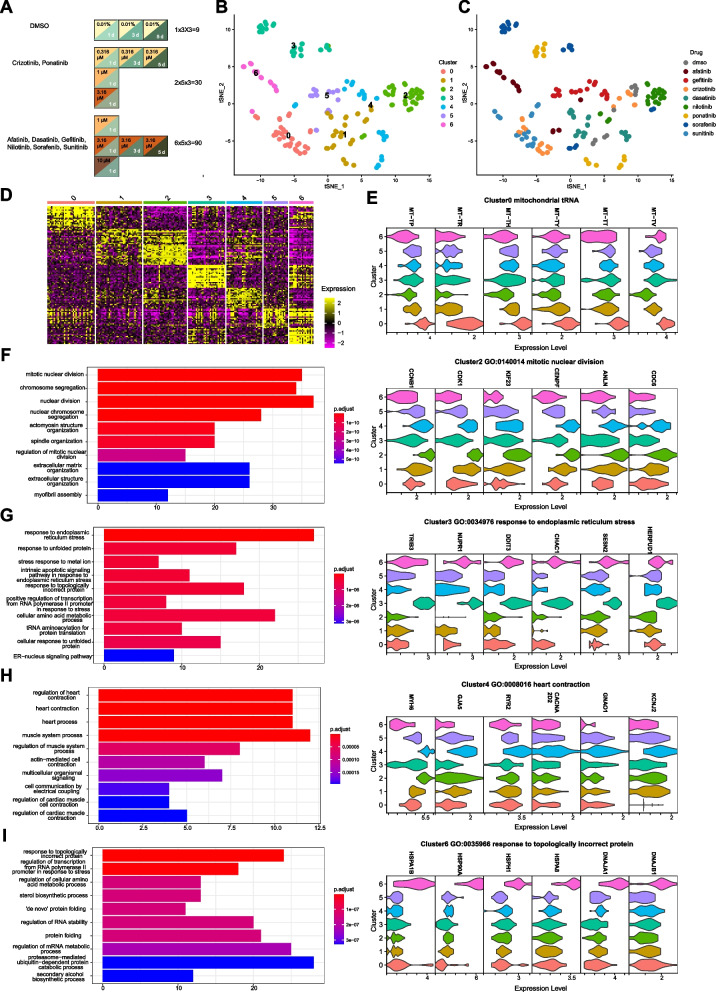
Major biological processes regulated by different TKIs. **A** The L- or T-shaped designs that span three doses and three time points for each TKI, selected based on results from Fig. 1A–H; 129 samples with three biological replicates per condition were measured in 3’DGE-UMI RNAseq. **B** Transcriptome changes induced by eight TKIs over dose and time were grouped into 7 clusters based on tSNE analysis, and the corresponding drugs of each cluster were shown in **C**. **D** Expression of top 20 gene markers for each cluster. **E** In Cluster 0, mitochondrial tRNA genes were expressed at a higher level than the other clusters. **F** Biological processes enriched for gene markers of Cluster 2. Expression of representative genes in GO term of mitotic nuclear division was shown on the right. **G** Biological processes enriched for gene markers of Cluster 3. Expression of representative genes in this GO endoplasmic reticulum stress was shown on the right. **H** Biological processes enriched for gene markers of Cluster 4. Expression of representative genes in GO term of heart contraction was shown on the right. **I** Biological processes enriched for gene markers of Cluster 6. Expression of representative genes in GO term of response to topologically incorrect protein was shown on the right

To further elucidate molecular mechanisms of cardiotoxicity induced by TKIs, we profiled transcriptome of hiPSC-CMs at different levels of toxicity in response to TKIs (the toxicity levels were determined based on the effects of TKIs on the ATP level, mitochondrial respiration, and beating properties). The transcriptome data followed a L- or T-shaped design (Fig. 2A). There were 129 samples in total with the vehicle controls at days 1, 3, and 5. To increase efficacy and reduce cost, we measured the transcriptome using the 3’digital gene expression with unique molecular identifiers (3’DGE-UMI) RNA-seq method [25, 38] where the UMI barcodes were used to label each drug treatment, rather than individual cells.

Based on tSNE analysis at a resolution of 2, drug-induced transcriptome responses were classified into 7 clusters (Fig. 2B). Clusters 2, 5, and 6 were composed of single drugs, nilotinib, gefitinib, and afatinib, respectively (Fig. 2B, C). Cluster 3 contained sorafenib and ponatinib; Cluster 0 was composed of crizotinib and sunitinib; Clusters 1 and 4 contained over three drug treatments (Fig. 2C). When the resolution was increased to 2.5 or 3 in the tSNE analysis, it did not change the overall clustering significantly, but split Cluster 0 and Cluster 3 into two clusters which mainly contained a single drug treatment (Fig. S4). As the variables of drug dose and treatment duration in the experimental design also affected transcriptional responses, the clustering separated most of drugs rather than each individual. Among the clusters, 0, 2, 3, 4, and 6 contained over 10 significantly differentially expressed genes (DEGs) that were used for gene ontology enrichment analysis (Figs. S5 and 2D). The primary biological process enriched in Cluster 3 was GO:0,034,976 response to endoplasmic reticulum stress and in Cluster 6 was GO: 0,035,966 response to topologically incorrect proteins (Fig. 2G, I). ER stress-related genes, including *HERPUD1, SESN2, CHAC1, DDIT3, NUPR1,* and *TRIB3*, were upregulated in both Clusters 3 and 6, but cytoplasmic chaperons, such as *DNAJB1* and *HSPA1B*, were only activated in Cluster 6. Cluster 3 was also enriched for tRNA aminoacylation of protein translation and the gene makers (such as *SARS *and* GARS*) were increased by both sorafenib and ponatinib (Figs. 2G and S6). Cluster 2 was enriched for mitotic nuclear division (Fig. 2F). Cluster 4 was enriched for genes associated with heart contraction, and these genes were upregulated by the treatments (gefitinib, dasatinib, and ponatinib at day 3 or day 5) (Fig. 2H). Even though not enriched, Cluster 0 was associated with higher expression of mitochondrial tRNA genes (such as *MT-TV, MT-TT,* and *MV-TY*, Fig. 2E). By comparing transcriptome changes induced by these drugs, we found that drugs with the same targets did not induce similar transcriptome changes, e.g., afatinib and gefitinib, dasatinib and nilotinib, sorafenib and sunitinib. Only sorafenib and ponatinib partially overlapped in Cluster 3. These results indicate that TKIs’ effect on transcriptome is drug-specific rather than target-specific.

Transcriptome data had good quality and consistency; 75 samples from day 1 treatments were barcoded twice to serve as technical replicates. In t-SNE plots, 75 technical replicates clustered closely (Fig. S7). The tSNE analysis was based on the top 10 principal components (PCs, *p* < 0.001, Fig. S8). About 10,000 unique genes and 10^5^ total counts were detected as median values for different samples (Fig. S9A, B). Percentage of mitochondrial DNA was high (10–50%) in our data, which is probably caused by mitochondrial damage of some TKIs [39, 40] (Fig. S9C). Additionally, the percentage of mitochondrial DNA negatively correlated with total RNA counts of each sample (correlation coefficient =  − 0.66 and *p* < 0.001, Fig. S10A), indicating that samples with mitochondrial damage had lower number of total reads measured. As expected, the number of unique genes detected positively correlated with total RNA counts (correlation coefficient = 0.89 and *p* < 0.001, Fig. S10B). When comparing the same drug treatments (sorafenib or sunitinib at 3.16 µM and 3 days) of the current 3’DGE RNA-seq with the published bulk RNAseq data (GEO GSE114686) [8, 41], we found that ~ 33.5% DEGs of sorafenib and ~ 38.4% DEGs of sunitinib overlapped; Pearson correlation was 0.91 for sorafenib and 0.87 for sunitinib (Fig. S11). In summary, transcriptome data revealed the major biological processes regulated by eight TKIs and ER stress was a shared response by three of them.

### Drug-specific effects on transcriptome dominate dose-, time-, or toxicity-induced effects

**Fig. 3:**
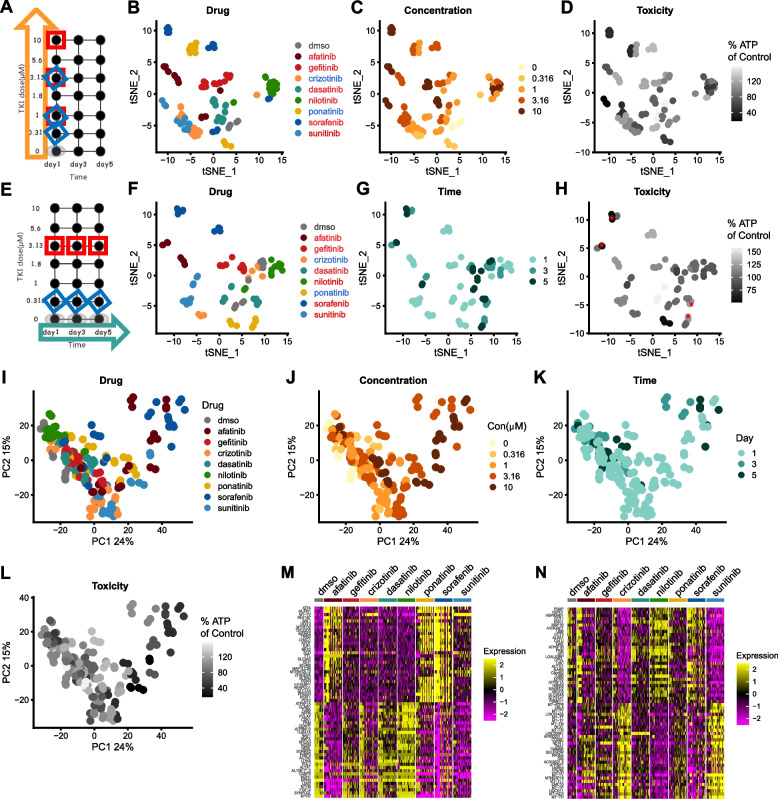
Drug specific effects on transcriptome dominate dose-, time- or toxicity-induced effects. **A** Transcriptome data were sliced to retain all drug treatments at day 1 with different doses. The orange arrow represents the concentration gradient of data. Red squares denote the doses for six TKIs (labeled in red in **B**) and blue diamonds denote the doses for two TKIs (labeled in blue in **B**). **B**–**D** Data selected as in **A** were projected into the tSNE space and shown with the properties of drug, concentration, or toxicity (represented by the ATP fold changes). Darker gray corresponds to higher toxicity. **E** Transcriptome data were sliced to retain drug treatments at a fixed dose over 5 days. The blue arrow represents the time gradient of data. Red squares denote the time points for six TKIs (labeled in red in **F**) and blue diamonds denote the time points for two TKIs (labeled in blue in **F**). **F**–**H** Data selected as in **E** were projected into the tSNE space and shown with the properties of drug, time or toxicity (represented by the ATP fold changes). Toxicity that was significantly different from controls was labeled with red asterisks in **H**. **I** Principal component analysis (PCA) of transcriptome changes induced by eight TKIs. **J** Drug concentration of each condition projected into the PCA space. **K** Treatment duration of each condition projected into the PCA space. **L** Toxicity level of each condition defined by the percent ATP of controls projected into the PCA space. Darker gray corresponds to higher toxicity. **M**–**N** Expression of genes with top and bottom 30 highest loadings of PC1 and PC2 grouped by drugs

To better visualize dose- or time-dependent effect of each drug, data were sliced and viewed based on either a concentration gradient or a time gradient (Fig. 3A, E). From the dose perspective, concentration was increased from the lower right to upper left direction for most drugs, except nilotinib, in the tSNE clustering (Fig. 3B, C). Afatinib, crizotinib, nilotinib, ponatinib, sorafenib, and sunitinib caused dose-dependent toxicity, whereas, dasatinib and gefitinib did not (Fig. 3B–D). When data were viewed longitudinally, afatinib, sorafenib, sunitinib, and dasatinib showed increasing toxicity with treatment duration, albeit to different degrees (Fig. 3E–H). Crizotinib, nilotinib, and ponatinib did not have time-dependent toxicity (Fig. 3F–H). When comparing changes from the dose and time gradients, four TKIs (afatinib, sorafenib, sunitinib, and dasatinib) showed similar directions of change in tSNE space; whereas three TKIs (ponatinib, crizotinib, and gefitinib) showed different, or even opposite, directions of change. In all the above cases, drug-specific effects on transcriptome dominated over dose-, time-, or toxicity-induced effects.

To analyze relation of TKI-induced transcriptome changes, principal component analysis (PCA) was run based on the top 2000 variable genes in the data. PC1 explained 24% of total variance, and PC2 explained 15% (Fig. 3I). Afatinib, sorafenib, and a few of ponatinib treatments were segregated from the other TKIs or vehicle controls from PC1 (Fig. 3I). This is consistent with the tSNE result where Cluster 3 (sorafenib and high-dose ponatinib-treated) and Cluster 6 (afatinib-treated) were segregated from the other TKIs (Fig. 2B, C). The PC2 axis distinguished crizotinib- and sunitinib-treated conditions, which were grouped into Cluster 0 in tSNE, from the rest of the drugs (Figs. 2B, C and 3I). Expression of genes with loadings ranked in the top 30 or the bottom 30 on PC1 or PC2 was plotted based on drugs (Fig. 3M, N). Consistent with Fig. 3I, the expression of genes with high PC1 loadings differentiated afatinib, sorafenib, and high-dose ponatinib from the rest of the drugs. Genes associated with ER stress (e.g., *ATF4, NUPR1, DDIT3, TRIB3, CHAC1, SESN2, HERPUD1*) were upregulated and genes related to cardiomyopathy and sarcomeric structure (e.g., *MYH7, SYNPO2L, LDB3, ACTN2, MYLK3*) were downregulated by afatinib, sorafenib, or high-dose ponatinib. Expression levels of high loading genes on PC2 distinguished the effect of crizotinib and sunitinib from the other TKIs (Fig. 3N). Among the high loading genes in PC2, genes associated with mitochondrial tRNAs (e.g., *MT-TH, MT-TP, MT-TL1, MT-TV*), as well as those function in mitochondrial electron transfer chain (e.g., *MT-ND3, UQCR11, COX7B, MTND3P19, ATP5IF1, COX6C*), were upregulated, similar to changes observed in gene markers of Cluster 0 (Figs. 3N and 2E). Consistent with the tSNE analysis, segregation of samples along PC1 or PC2 was dependent on drug type, rather than concentration, treatment duration, or toxicity (Fig. 3I–L). Therefore, transcriptome data revealed drug-specific, concentration-, and time-dependent responses in human cardiomyocytes to TKIs, with the drug-specific effect as the dominating factor.

### Afatinib, sorafenib, and ponatinib induce ER stress in rat cardiomyocytes

**Fig. 4:**
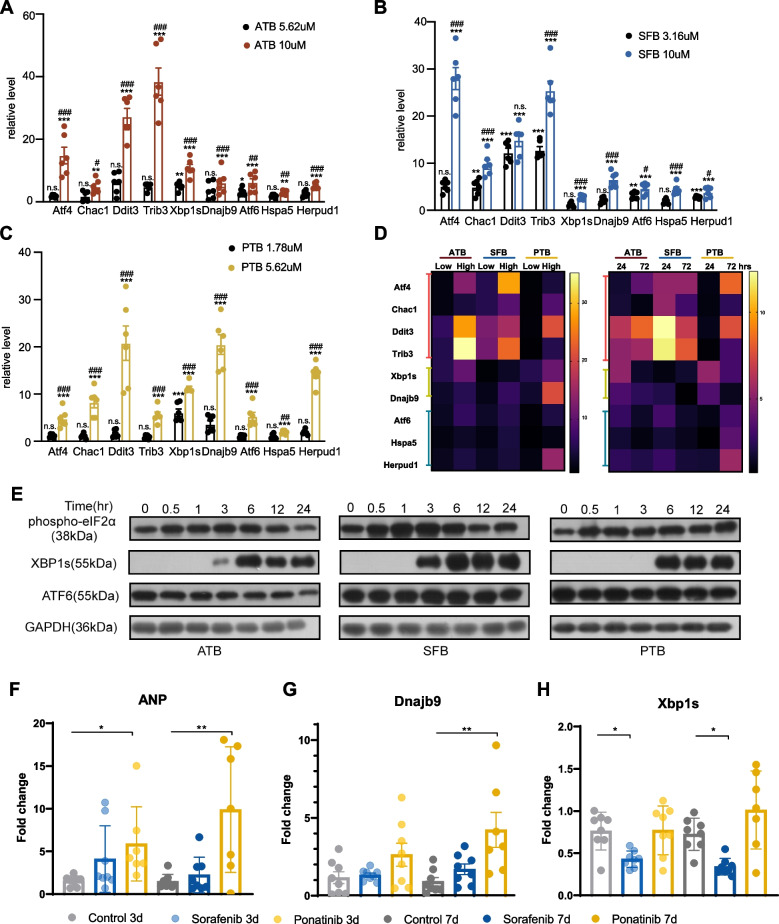
Afatinib, sorafenib and ponatinib induce ER stress in NRCMs and rat hearts. **A** Fold changes of ER stress related genes, Atf4 and its targets, Chac1, Ddit3, Trib3;Xbp1s, and its target Dnajb9; Atf6 and its targets Hspa5 and Herpud1, in NRCMs treated with afatinib at 5.62 or 10 µM for 24 h. **B** Fold changes of ER stress related genes in NRVMs treated with sorafenib at 3.16 or 10 µM for 24 h. **C** Fold changes of ER stress related genes in NRCMs treated with ponatinib at 1.78 or 5.62 µM for 24 h. **D** Left: heatmap of gene expression changes from **A**–**C**. Right: heatmap of gene expression changes caused by afatinib 5.62 µM, sorafenib 3.16 µM, or ponatinib 1.78 µM at 24 or 72 h. **E** Phospho-eIF2α (or EIF2S1), XBP1s, ATF6 (cleaved), and GAPDH expression at different time points under afatinib, sorafenib, and ponatinib treatment measured by Western blot. **F**–**H** Fold changes of Anp, Dnajb9, and Ddit3 in rat left ventricles from sorafenib or ponatinib gavage for 3 or 7 days. **A-C** Data were presented as mean ± SEM (*n* = 3) and analyzed using ANOVA analysis. *p < 0.05, **p < 0.01, ***p < 0.001 versus the DMSO vehicle control group. ^#^*p* < 0.05, ^##^*p* < 0.01, ^###^*p* < 0.001 versus the lower dose. **F**–**H** Data were presented as mean ± SEM (*n* = 8) and analyzed using ANOVA analysis. *p < 0.05, **p < 0.01, ***p < 0.001 versus the vehicle control

Each batch of hiPSC-CMs took about a month to differentiate and were very expensive to culture or purchase. Neonatal rat cardiac myocytes (NRCMs) took 1 day to isolate from neonatal rat hearts and were less expensive to culture; therefore, we chose NRCMs over hiPSC-CMs for the subsequent mechanistic studies. Admittedly, NRCMs were derived from rat and could have species differences from human, but these cells had been widely used to study molecular mechanisms of cardiac hypertrophy and provided translatable findings related to cardiac diseases [42]. Additionally, NRCMs shared similar sensitivity to afatinib, sorafenib, and ponatinib with hiPSC-CMs (Fig. S12). So, we used NRCMs to validate the upregulation of ER stress observed in previous RNA-seq data. NRCMs were treated with afatinib at 5.62 or 10 μM, sorafenib at 3.16 or 10 μM and ponatinib at 1.78 or 5.62 μM for 24 h and with the lower doses of the three TKIs for 72 h. Gene targets of three ER stress effectors, Atf4, Xbp1s, and Atf6, were upregulated by the three TKIs at high doses and 24 h (*p* < 0.001, Fig. 4A–C). Chac1, Ddit3, and Trib3 are gene targets of Atf4. Consistent with upregulation of Atf4, Chac1, Ddit3, and Trib3 were upregulated by the three TKIs at high doses and 24 h (*p* < 0.01, Fig. 4A–C). Dnajb9 is a downstream target of Xbp1s. Ponatinib treatment caused the most robust upregulation of Dnajb9 among the 3 TKIs (*p* < 0.001, Fig. 4A–C). Hspa5 and Herpud1 are downstream targets of Atf6 (Hspa5 is also up-regulated by Atf4). Herpud1 was most induced by ponatinib (*p* < 0.001, Fig. 4A–C). Hspa5 was increased similarly by the three TKIs (*p* < 0.01, Fig. 4A–C). Despite the drug- and dose-dependent increase in the expression of ER stress genes, activation of three UPR branches was biased. With high-dose treatment, afatinib and sorafenib activated the ATF4 axis more robustly than the XBP1s and the ATF6 axes, while ponatinib induced activation of three axes to similar degrees (Fig. 4D, left). The three TKIs induced different temporal activation of ER stress genes. Afatinib caused a higher expression of Atf4, but a lower expression of Xbp1s and Atf6 at 72- than 24-h treatment (Figs. 4D, right, and S13A). For sorafenib at 3.16 μM, most gene targets of ER stress were downregulated over time, indicating a transient ER stress response (Fig. 4D, right, and S13B). For ponatinib treated at 1.78 μM, the ATF4 and the ATF6 axes, but not XBP1s, showed time-dependent upregulation (Fig. 4D, right, and S13C). In summary, afatinib, sorafenib, and ponatinib not only induced ER stress at high doses acutely but also at low doses chronically.

We further explored the temporal activation of ER stress at protein level in response to the 3 TKIs. Phosphorylation of eIF2α (gene name: *Eif2s1*), which is downstream of PERK and upstream of Atf4, responded quickly to the 3 TKIs and was activated within 30 min (Fig. 4E). Ponatinib activated phosphorylation of eIF2α for 24 h, but afatinib and sorafenib activated it for 12 or 6 h, respectively (Fig. 4E). XBP1s was activated much later than eIF2α, after 3 or 6 h of the TKI treatment and remained elevated for 24 h (Fig. 4E). The expression of activated-ATF6 was not changed by any drug treatment (Fig. 4E). To validate that these TKIs also induce the expression of ER stress genes in human cardiomyocytes, we analyzed the public dataset GSE114686. The data were based on treating Cor.4U hiPSC-CMs, namely the same cells used in the current study, with four TKIs (only sorafenib was overlapped with the current study). In GSE114686, gene markers of ER stress (including *ATF4, CHAC1, DDIT3, TRIB3, XBP1, DNAJB9, ATF6, HSPA5, HERPUD1*) were all upregulated by sorafenib at 10 µM for 24 h or at 3.16 µM for 168 h (see Table S2), supporting that ER stress induced by sorafenib also happens in human cardiomyocytes and is probably independent of the species of cell models.

### Sorafenib and ponatinib modulate ER stress in adult rat hearts

To validate TKI-induced ER stress in vivo, we established cardiotoxicity animal models by gavaging Sprague–Dawley rats with either ponatinib (15 mg/kg) or sorafenib (50 mg/kg) once daily (see Table S3 The ARRIVE checklist). We did not do it for afatinib because this drug was not reported highly cardiotoxic. The dose of the drugs was selected based on literature [43, 44], as well as human-to-rat dose conversion based on body surface area principle. As ER stress is usually a transient response, we reasoned that we should collect the hearts at early time points before obvious cardiotoxicity. Rats were gavaged for 3 or 7 days and the hearts were collected to measure gene markers of ER stress. Ponatinib treatment was more toxic than sorafenib as one rat died in the ponatinib-treated group. Heart or body weight was not changed by sorafenib, but decreased by ponatinib at day 7 (*p* < 0.01 and *p* < 0.001, respectively, Fig. S14A–C). The ratio of heart weight to body weight was not changed in any treatment. Ponatinib induced a significant increase in gene expression of Anp (official gene name: *Nppa*), which is a fetal gene re-expressed during cardiac stress or dysfunction, indicating that potential cardiotoxicity was induced by this drug (*p* < 0.01, Fig. 4F). Sorafenib did not induce a significant change in Anp, but 2–3 animals had a high fold increase in Anp expression and this reflected variation in drug response among individual rats (Fig. 4F). Consistent with the cardiotoxicity induced by ponatinib, Dnajb9 expression was significantly increased at day 7, indicating the activation of Xbp1s axis of ER stress (*p* < 0.01, Fig. 4G). Sorafenib caused a significant reduction of Xbp1s (*p* < 0.05, Fig. 4H), the consequences of which need further exploration. Neither drugs caused a significant change in Ddit3, which is downstream of the PERK-peIF2α-Atf4 pathway (Fig. S14D).

### Afatinib, sorafenib, and ponatinib induce different levels of lipid peroxidation, ROS, calcium defects, and TNNT2 loss in rat cardiomyocytes

**Fig. 5:**
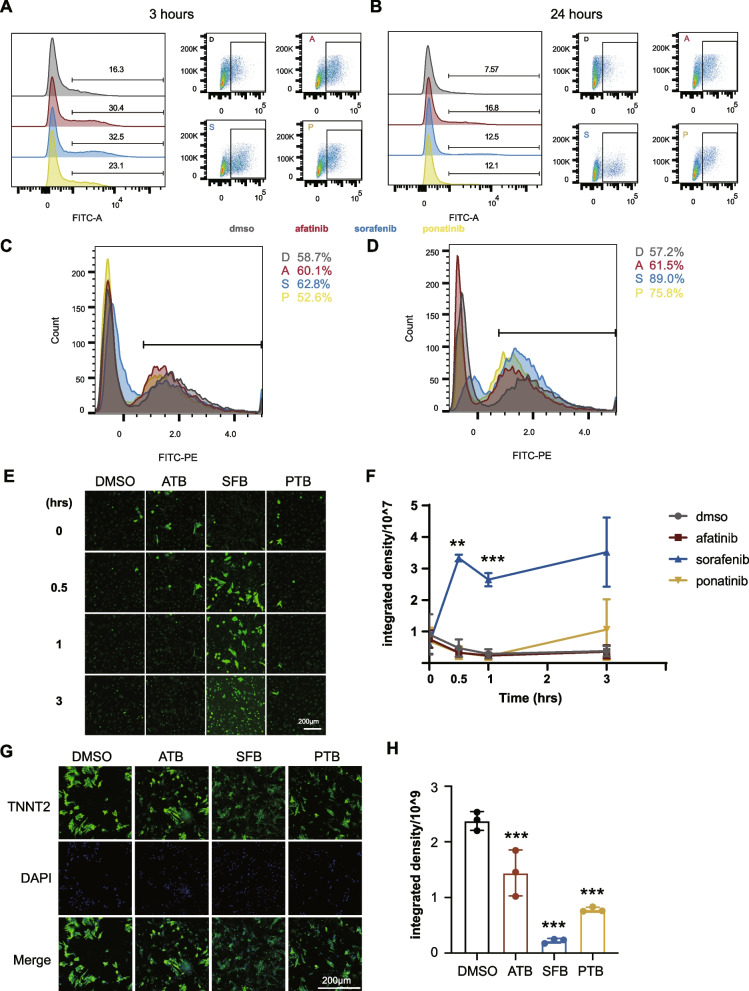
The 3 TKIs induce different levels of lipid peroxidation, ROS, calcium defects, and TNNT2 loss in NRCMs. To measure oxidative stress and cardiotoxic effects induced by TKIs, NRCMs were treated with 10 µM afatinib, 10 µM sorafenib, or 5.62 µM ponatinib and stained with various dyes before quantification by flow cytometry or imaging. **A-B** Percent of ROS-high cells quantified by flow cytometry and staining with H_2_DCFDA after different TKI treatments for 3 or 24 h. **C–D** Histogram of the ratios between green (oxidized) and red (non-oxidized) fluorescence intensity of C11-bodipy^581/591^ in NRCMs under different treatments. Percentages by the condition names were the fraction of cells in the specified gate for each treatment. **E** Representative fluorescence images of live NRCMs pre-loaded with Calbryte™ 520 AM and treated with the indicated drug from 0 to 3 h. **F** Integrated density of green fluorescence in **E**. **G** Representative immunofluorescence images of TNNT2 (green) and nuclei (blue) in NRCMs treated with TKIs for 24 h. **H** Integrated density of green fluorescence in **G**. Data were presented as mean ± SEM (*n* = 3) and analyzed using ANOVA analysis. *p < 0.05, **p < 0.01, ***p < 0.001 versus the DMSO vehicle control group. Scale bar: 200 µm

Oxidative stress is an inducer of ER stress [45], so we also evaluated whether these TKIs induce reactive oxygen species (ROS) in NRCMs by flow cytometry. All three TKIs induced an increase in free ROS at 3 h of treatment. Specifically, the percent of ROS-high cells was increased by 14.1%, 16.2%, and 6.8% by afatinib, sorafenib, and ponatinib, respectively (Fig. 5A). For sorafenib treatment, the median fluorescence intensity of the ROS-high cells was about twofolds of that in the control group. These TKIs also induced an increase in ROS at 24 h, but the percent of ROS-high cells was lower than that at 3 h (Fig. 5B), indicating that ROS induction may be transient. Lipid peroxidation measured by the ratio of fluorescence intensity from the FITC channel to the PE channel was also assessed at 3 and 24 h. At 3 h of treatment, the percent of cells with high lipid peroxidation was increased slightly by afatinib and sorafenib (Fig. 5C). However, at 24 h of treatment, the three TKIs increased the percent of cells with high lipid peroxidation by 4 to 32% (Fig. 5D); the level induced by sorafenib was the closest to that of the positive control, cumene hydroperoxide (42%, Fig. S15). Ca^2+^ also plays a vital role in maintaining ER homeostasis and cardiomyocyte contraction [46], and calcium overload is associated with ER stress and contraction/relaxation defects. So we measured how these TKIs affect calcium homeostasis of NRCMs. Sorafenib induced a significant cytosolic overload of Ca^2+^ in NRCMs at 0.5 and 1 h (*p* < 0.01 and *p* < 0.001, respectively); whereas afatinib and ponatinib did not cause any significant changes in calcium concentration (Fig. 5E, F). We then tested whether antioxidants, such as trolox, can rescue the ER stress induced by these TKIs. While trolox reduced the level of lipid peroxidation induced by afatinib and sorafenib at 24 h (92.5% vs 83% and 94.6% vs 88%), it did not block the induction of ER stress by these TKIs (Fig. S16). Paradoxically, the combination of afatinib or sorafenib with trolox increased the expression of some ER stress genes compared with TKI alone (Fig. S16). As ER stress is dynamic, it could be that the anti-oxidant extended the activation duration of ER stress or that some other factors regulated ER stress cooperatively with oxidative stress. TNNT2 protein (or cardiac Troponin T2), a sarcomeric component and marker for cardiac damage, was inhibited by the 3 TKIs, among which sorafenib had the most effect (*p* < 0.001, Fig. 5G, H). In summary, ROS level and lipid peroxidation were increased by the three TKIs, and calcium overload induced by sorafenib prior to ER stress. The toxicity of these TKIs was also associated with a decrease in cardiac Troponin T2 protein expression, which may negatively affect myocardial cell contraction.

### Afatinib, sorafenib, and ponatinib cause increased expression of fetal and pro-inflammatory genes through coordinated activation of PERK and IRE1α pathways

**Fig. 6:**
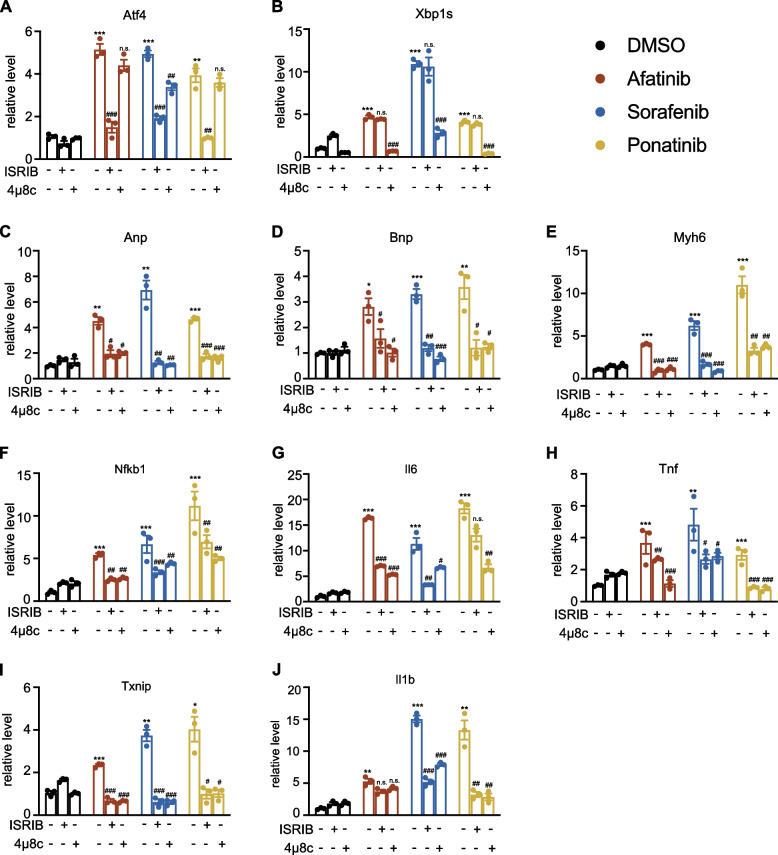
The 3 TKIs cause increased expression of fetal and pro-inflammatory genes through coordinated activation of PERK and IRE1α pathways. **A–J** Fold changes of Atf4, Xbp1s, Anp, Bnp, Myh6, Nfkb1, Il6, Tnf, Txnip, and Il1b mRNAs in NRCMs treated with 3 TKIs (10 μM afatinib, 10 μM sorafenib, 5.62 μM ponatinib) in combination with or without ISRIB (200 nM) or 4μ8c (10 μM) for 24 h. Data were presented as mean ± SEM (*n* = 3) and analyzed using ANOVA analysis. *p < 0.05, **p < 0.01, ***p < 0.001 versus the DMSO vehicle control group. ^#^*p* < 0.05, ^##^*p* < 0.01, ^###^*p* < 0.001 versus the TKI treatment alone

ER stress could cause cardiac damage by inducing apoptosis or inflammation through one or combinations of three effector pathways (PERK-ATF4, IRE1α-XBP1s, or ATF6). To evaluate which pathway(s) of ER stress play an important role in TKI-induced cardiomyocyte injury, we treated NRCMs with pathway-selective inhibitors and measured gene expression and cell viability. Integrated stress response inhibitor (ISRIB) was used to inhibit PERK-ATF4 signaling and 4μ8c (IRE1 Inhibitor III) was used to inhibit IRE1α-XBP1s axis. To validate the effect of these inhibitors, we measured the expression of Atf4 and its target Ddit3, Xbp1s, and its target Dnajb9, respectively. Consistent with the specificity of these drugs, ISRIB inhibited Atf4 and Ddit3 significantly without affecting the XBP1s axis too much, meanwhile 4μ8c inhibited Xbp1s and Dnajb9 without affecting the ATF4 axis (*p* < 0.05 at least, Fig. 6A, B, Fig. S17).

When PERK or IRE1α was inhibited by pathway-selective inhibitors, cell viability was not significantly changed with co-treatment of TKIs and ISRIB or 4μ8c (Fig. S17A–C). In terms of cell death, afatinib and ponatinib induced very little cell death after 24-h treatment, but sorafenib induced a lot of cell deaths (*p* < 0.001, Fig S18). 4μ8c (inhibitor of Xbp1s) significantly rescued cardiac cell from death induced by sorafenib (*p* < 0.001), but the inhibition of PERK axis had no such effect (Fig. S18). The data suggested that neither downstream effectors of ER stress induced by afatinib or ponatinib was associated with cell death, but IRE1α-XBP1s activation was required for sorafenib-induced cell death, albeit partially.

Besides, we explored whether the 3 TKIs activated inflammation downstream of ER stress in NRCMs. Inflammation-related genes, such as Nfkb1, Il-6, Tnf, Txnip, and Il1b, were increased significantly after 24-h treatments of the 3 TKIs (from *p* < 0.05 to *p* < 0.001, Fig. 6F–J). Induction of all these inflammatory genes, as well as the cardiac fetal genes (Anp, Bnp/Nppb, and Myh6), was blunted by 4μ8c when used as a co-treatment with TKIs (Fig. 6F–J). Similarly, ISRIB inhibited the expression of cardiac fetal genes to a similar level as 4μ8c (Fig. 6C–E). ISRIB also inhibited the expression of Txnip, Il1b, and Tnf to similar degrees as 4μ8c, but did not hamper the expression of Nfkb1 and Il6 as potently as 4μ8c in combination with ponatinib (Fig. 6F–J). These results suggested that both PERK and IRE1α downstream of ER stress are required for the induction of pro-inflammatory and cardiac fetal genes by TKIs. Gene markers of inflammasomes, Txnip and Il1b, were regulated in a similar manner by TKIs with or without ER stress inhibitors; whereas Nfkb1, Tnf, and Il6 of a different inflammatory pathway were regulated similarly (Fig. 6F–J). Similar changes in gene expression were observed in the H9C2 cell line (Fig. S19). Inhibiting phosphorylation of eIF2α by ISRIB blocked upregulation of inflammatory genes (Nfkb1, Il6. and Tnf) by TKIs. However, inhibition of dephosphorylation of eIF2α with salubrinal increased the expression level of some pro-inflammatory genes, such as Nfkb1, Il6, and Tnf, but not Il1b (Fig. S19). In summary, afatinib, sorafenib, and ponatinib cause re-expression of cardiac fetal genes and promote pro-inflammatory gene expression through PERK-peIF2α-ATF4 and IRE1α-XBP1s pathways.

## Discussion

Many TKIs have transformed cancers into chronic diseases with extended drug therapy; however, TKI-induced cardiotoxicity becomes a significant problem to cause death or decrease life quality. Treatment that reduces cardiac damage caused by TKIs is lacking due to limited understanding of molecular pathology. Many TKIs have multiple targets besides receptor tyrosine kinases and could cause cardiotoxicity through non-signaling mediated effects, such as transcriptomic regulation. Here, we discover enriched biological processes regulated transcriptionally by eight TKIs in hiPSC-CMs as candidates for their cardiotoxic mechanisms and validate that ER stress-induced inflammation is a common mechanism of cardiotoxicity induced by three TKIs in cardiomyocytes. TKI-specific transcriptome changes dominate over dose- and time-dependent effects. Three TKIs, afatinib, sorafenib, and ponatinib, induce ER stress in cardiomyocyte and ponatinib also increases the IRE1α-XBP1s pathway and fetal gene expression in rat left ventricles. Afatinib, sorafenib, and ponatinib induce expression of fetal and pro-inflammatory factors in cardiac myocytes through cooperative activation of PERK and IRE1α. Through validating the critical role of ER stress in regulating cardiotoxicity of many TKIs, our study suggests that transcriptome data can direct us to find important molecular pathology of cardiotoxicity. We also pinpoint specific pathways downstream of ER stress that can serve as potential treatments for TKI-induced cardiotoxicity.

Predicting cardiotoxicity of drugs using in vitro assays remain challenging and researchers try to improve the accuracy of prediction by increasing the number of cell lines or the types of measurements. A traditional assay to evaluate cardiotoxicity is testing the inhibitory effect on the inward rectifying potassium channel (hERG), yet it is criticized for a high false positive rate (36%) [47] and a non-physiological cell type. The model of hiPSC-CMs is a promising cell type that preserves not only electrophysiology but also metabolic and contractile phenotypes of cardiomyocytes. So, it is proposed to serve as a new platform for in vitro cardiotoxicity screening (see the CiPA initiative [48]). We use this cell type to do both phenotypic and transcriptomic analysis in the hope to find the most relevant changes to human and help improve the correlation between in vitro data and patient-observed cardiotoxicity. When we rank the toxicity of TKIs based on different cellular assays and patient-derived data, we find that the toxicity of the eight TKIs measured in cellular assays mimics that observed in FAERS or literature. Three out of five drugs with high toxicity, sunitinib, sorafenib, and ponatinib, are shared between FAERS/literature and cellular assays. Additionally, the toxicity of the TKIs on cell viability is similar to that observed in cellular contraction and electrophysiology measurement, which is consistent with a previous study [49]. Afatinib is ranked as highly cardiotoxic based on mitochondrial and beating assays, with little toxicity found in FAERS or literature; this discrepancy could be due to that the dose of afatinib used in vitro (10 µM) is much higher than its Cmax (0.05 µM) in vivo*.* But our results suggest that patients who cannot metabolize afatinib properly or timely may experience unduly cardiotoxicity. Dasatinib is ranked as highly cardiotoxic based on FAERS and literature, but does not affect the functions of cardiomyocytes significantly in vitro, indicating that dasatinib may target other cell types or other organ systems to negatively affect cardiovascular function. Different phenotypic assays put weights on different aspects of cardiomyocyte function, for example, the seahorse assay measures energy metabolism and the microelectrode array measures contractility, so it is hard to combine the results into a single risk factor in a simple way. By evaluating a larger number of drugs with different levels of cardiotoxicity and focusing on specific phenotypes (cardiac arrhythmia as in CiPA or heart failure), we may be able to construct an efficient in vitro assay with appropriate algorithms to consider different phenotypic data in order to predict cardiotoxicity.

Besides phenotypic data, transcriptomic changes can also contribute to cardiotoxicity risk stratification of TKIs. However, current transcriptome data do not stratify TKIs according to toxicity, but based on drug identity. Most drugs elicit distinct transcriptomic changes; only sorafenib and ponatinib or sunitinib and crizotinib share similar transcriptional signatures. While the similarity between sorafenib and ponatinib can be explained by shared targets, that between sunitinib and crizotinib remains unknown. How to utilize the transcriptomic signatures for in vitro cardiotoxicity evaluation requires deeper understanding of the role of these signatures in cardiotoxicity. To explore this, we focus on transcriptional changes of a stress response pathway that is repeatedly identified in dimension reduction and enrichment analyses. Both tSNE and PCA analyses show that afatinib, sorafenib, and ponatinib induce the most distinct transcriptomic changes from the other TKIs and the changes unify on ER stress activation. Afatinib and sorafenib primarily activate PERK and IRE1α, whereas ponatinib activates all three effectors of ER stress in NRCMs. Ponatinib also upregulates the IRE1α-XBP1s pathway in rat hearts, but sorafenib shows opposite regulation of this pathway in vivo versus in vitro. This could be due to different temporal activation of the ER stress, different cross-regulatory mechanisms or many more cell types present in vivo versus in vitro, and needs further clarification. Inhibition of ER stress pathways blocks the induction of fetal or proinflammatory genes by TKIs, suggesting that ER stress promotes cardiotoxicity in cardiomyocytes. Even though all three TKIs activate ER stress, afatinib has a much lower cardiotoxicity reported in clinic than sorafenib or ponatinib. The reason may be that afatinib’s EC50 on cells is ~ 60 folds higher than its *C*_max_ in vivo. Another possible reason may be that afatinib induces many compensatory pathways to reverse cardiotoxicity, such as the activation of cytosolic chaperons and the Nrf2 pathway [50, 51], which are not induced by sorafenib or ponatinib in cardiomyocytes.

In searching for effective treatment to reduce cardiotoxicity without affecting the therapeutic effect of drugs, one needs to validate that the mechanism of cardiotoxicity is cardiac specific or have differential effects in the tumor versus in the heart. For example, if ER stress promotes cardiotoxicity but is dispensable for or even inhibits cancer killing by TKIs, this process may be a promising target for developing oncocardiology treatment. To compare the effect of ER stress in cardiomyocytes and cancer cells, we searched literature about TKI-induced ER stress in cancer cells. Afatinib induces the activation of the PERK-eIF2α pathway downstream of ER stress in the head and neck squamous cell carcinoma and sorafenib induces both PERK and IRE1α activation in leukemia and hepatocellular carcinoma cells [52–54]. So ER stress is activated in both cardiomyocytes and cancer cells. Then, what is the role of ER stress in the therapeutic effect of these TKIs? In the case of afatinib, ER stress activation is required for drug-induced apoptosis [52]. So ER stress inhibition may negatively affect afatinib’s therapeutic effect, thus not desirable for treating the associated cardiotoxicity. In the case of sorafenib, the role of ER stress in drug-induced apoptosis in cancer is still debatable. One study shows that blockade of IRE1α or PERK can enhance apoptosis induced by sorafenib [53], whereas another study finds that enhancing ER stress increases apoptosis and inhibits tumor growth in vivo in response to sorafenib [54]. ER stress also promotes drug resistance to sorafenib through various mechanisms in HCCs [55–57]. Therefore, whether ER stress plays a positive or negative role in the anti-cancer therapeutic effect of sorafenib needs further clarification. Besides existing reports, to validate ER stress as a worthwhile target to treat cardiotoxicity without negatively affecting tumor treatment, one needs to carry out more experiments using both cells and tumor-bearing animal models.

ER stress is usually adaptive initially and causes detrimental effects if persisted. Upon afatinib, sorafenib, and ponatinib treatment, the initial induction of ER stress is probably beneficial for cardiac cells to restore ER homeostasis. However, if the repair attempts fail and the ER stress persists, this may lead to cellular damages, such as apoptosis or inflammation. ER stress induced by afatinib or ponatinib does not cause apoptosis, while that by sorafenib causes mild degree of apoptosis in cardiomyocytes. Yet the TKI-induced ER stress upregulates inflammation, including the inflammasome-IL1β pathway and the NF-κB pathway, in cardiomyocytes. Like the known pathways in immune cells [58, 59], ER stress promotes expression of pro-inflammatory genes in cardiomyocytes, albeit dependent on coordinated activation of both PERK and IRE1α. Since inflammatory factors, such as IL1β and IL6, increase risk of cardiovascular diseases [60, 61], the signaling from ER stress to inflammation induced by the TKIs may be a potential target for treating cardiotoxicity. Our finding is consistent with previous ones that sorafenib induces inflammation in skin [62] and ponatinib causes inflammation in endothelial cells [63] and in the ischemic brain of zebrafish [64]. Since production of pro-inflammatory factors can exacerbate ER stress, whether inflammation is just a consequence of ER stress or it modulates ER stress and cardiotoxicity to TKIs still needs further investigation. As the three effectors of ER stress have adaptive and maladaptive functions depending on the duration and the selected pathways activated, maximizing the adaptive and minimizing the maladaptive functions of the ER stress may be an effective way to mitigate the cardiotoxicity that is caused by this pathway.

Our study is limited in many aspects. First, TKI-induced cardiotoxicity can be regulated by non-ER stress mechanisms that are not evaluated in the current study. For example, sorafenib inhibits mitochondria, induces ROS accumulation, and regulates metabolism of cardiomyocytes [8, 44, 65]. How ER stress is associated with other pathways that regulate cardiotoxicity remain to be determined for many of the TKIs. Secondly, we established animal models based on short-term treatments of two cardiotoxic TKIs (ponatinib and sorafenib) and did not measure cardiac function, as the short-term intervention should not cause a noticeable change in cardiac function. It is worthwhile to treat the animals for a comparable duration with that of human therapy, or at least a month as generally done in literature, and measure ER stress at different time points over the course, as well as cardiac function at the end time point. Thirdly, we have not identified the upstream trigger of ER stress by TKIs. We tried to evaluate the role of ROS in ER stress activation, but our data suggest a paradoxical role of ROS. The antioxidant trolox, used in this paper, has a mild effect in inhibiting ROS or lipid peroxidation induced by TKIs, and the time points measured are limited. So, further study using some other antioxidants and at more time points is warranted. Last but not least, due to the high cost and long duration of using hiPSC-CMs, we switched to use NRCMs for mechanic studies. Our data and literature suggest that the two cell models agree in most of the phenotypic and molecular assays. To avoid species-related difference, one should further validate the ER stress-associated signaling pathways and gene expression induced by TKIs in more hiPSC-CMs or primary human cardiomyocytes.

## Conclusions

Afatinib, sorafenib, and ponatinib induce ER stress, pro-inflammation, and cardiac fetal gene expression to cause cardiotoxicity. Inhibition of either PERK or IRE1α axes of the ER stress pathway blocks the expression of cardiac fetal and pro-inflammatory genes. This mechanism is a potential therapeutic target to mitigate sorafenib- and ponatinib-induced cardiotoxicity. Our study also elucidates important biological processes regulated by some other TKIs that should be further explored and translated into in vitro cardiotoxicity prediction or cardio-oncology treatment.

## Supplementary Information

**Additional file 1: Table S1.** An introduction to the pharmacology and toxicology of TKI drugs of different cardiotoxicity levels. **Table S2.** Endoplasmic reticulum stress gene markers under different conditions of sorafenib. **Table S3.** The ARRIVE checklist.

**Additional file 2: Figure S1.** EC50s of response calculated based on a four-parameter log-logistic model in the ATP fold change, related to Fig. 1. **Figure S2.** Seahorse experiment on acute effects of TKIs on mitochondrial oxygen consumption and extracellular acidification, related to Fig. 1. **Figure S3.** Mitochondrial membrane potential changes in response to TKIs observed by TMRE staining and its fold change, related to Fig. 1. **Figure S4.** Clustering of TKI-induced transcriptome data based on tSNE analysis, related to Fig. 2. **Figure S5.** Cluster 0, 2, 3, 4, 6 contained over 10 significant DEGs found by log2-based fold changes, related to Fig. 2. **Figure S6.** Expression of genes related to tRNA aminoacylation for protein translation in different clusters or in response to different drugs, related to Fig. 2. **Figure S7.** Good quality and consistency of 3’DGE-UMI RNA-seq, related to Fig. 2. **Figure S8.** The Jackstraw plot of the top 15 principal components in the tSNE analysis, related to Fig. 2. **Figure S9.** The number of unique genes, total counts, and proportion of mitochondrial DNA present in the 3'DGE-UMI RNA-seq data, related to Fig. 2. **Figure S10.** Correlation analysis between mitochondrial DNA and total counts or between unique genes and total counts in 3’DGE-UMI RNA-seq data, related to Fig. 2. **Figure S11.** Comparison of differentially expressed genes detected by 3'DGE-UMI and bulk RNA-seq for sorafenib and sunitinib treatments, related to Fig. 2.

**Additional file 3: Figure S12.** Cell viability of NRCMs in response to afatinib, sorafenib, and ponatinib, related to Fig. 4. **Figure S13.** Low dose of TKIs induce ER stress over time in NRCMs, measured by mRNA fold changes, related to Fig. 4. **Figure S14.** Effects of ponatinib and sorafenib on heart weight, body weight, heart-to-body weight ratio, and Ddit3 expression in rat hearts, related to Fig. 4. **Figure S15.** Lipid peroxidation levels in NRCMs treated with cumene hydroperoxide and ethanol, related to Fig. 5. **Figure S16.** The effect of trolox on lipid peroxidation and ER stress induced by TKIs, related to Fig. 5. **Figure S17.** ISRIB and 4μ8c affected gene targets of Atf4 and Xbp1s induced by TKIs in NRCMs, related to Fig. 6. **Figure S18.** ISRIB and 4μ8c did not rescue NRCMs from cell death induced by the 3 TKIs, related to Fig. 6. **Figure S19.** The effects of ISRIB and 4μ8c on TKI-induced cell death examined using fluorescence imaging and quantification, related to Fig. 6. **Figure S20.** Persistent eIF2α phosphorylation up-regulated Nfkb1 and Il6 expression induced by 3 TKIs in H9C2 cells, but not Il1b or Tnf, related to Fig. 6.

**Additional file 4.** Supplementary research methods and details on the reagent or resource used in the experiment.

**Additional file 5.** Original western blot gel images, related to Fig. 4.

## Data Availability

The datasets generated and/or analyzed during the current study are available upon request to the corresponding author. The original RNAseq data were deposited in the GEO database with the accession number of GSE221595 (this data will be made public upon the publication of this paper).

## Abbreviations

3’DGE-UMI: 3’Digital gene expression with unique molecular identifiers
ATB: Afatinib
CHOP: C/EBP homologous protein
EC50: Concentration for 50% of maximal effect
ER: Endoplasmic reticulum
FAERS: FDA adverse event reporting systems
FBS: Fetal bovine serum
hiPSC-CMs: Human induced pluoripotent stem cell-derived cardiomyocytes
IR: Schemia/reperfusion
IRE1α: Inositol-requiring enzyme 1α
ISRIB: Integrated stress response inhibitor
NRCMs: Neonatal rat cardiomyocytes
PERK: Protein kinase R like endoplasmic reticulum kinase
PTB: Ponatinib
ROS: Reactive oxygen species
SFB: Sorafenib
TKIs: Tyrosine kinase inhibitors
tSNE: T-Distributed Stochastic Neighbor Embedding
XBP1: X-box binding protein 1
