# Supplementary material for: Three tyrosine kinase inhibitors cause cardiotoxicity by inducing endoplasmic reticulum stress and inflammation in cardiomyocytes
Source: BMC Med. 2023 Apr 17;21:147. doi: 10.1186/s12916-023-02838-2 (PMC10108821; doi:10.1186/s12916-023-02838-2)

**Three tyrosine kinase inhibitors cause cardiotoxicity by inducing endoplasmic reticulum stress and inflammation**

Huan Wang^1,#,*^, Yiming Wang^1,#^, Jiongyuan Li^1^, Ziyi He^1^, Sarah A. Boswell^2^, Mirra Chung^2^, Fuping You^1^, Han Sen^3^

1. Institute of Systems Biomedicine, School of Basic Medical Sciences, Peking University Health Science Center, Beijing, 100191, China

2. Laboratory of Systems Pharmacology, Department of Systems Biology, Harvard Medical School, Boston, Massachusetts 02115, USA

3.Key Laboratory of Carcinogenesis and Translational Research (Ministry of Education), Peking University Cancer Hospital & Institute, Beijing 100142, China.

* Corresponding author, email: [huan_sharon_wang@pku.edu.cn](mailto:huan_sharon_wang@pku.edu.cn)

# these authors contributed equally

| **TKI** | **Inhibitor Target** | **Primary FDA Indication** | **Dose**  **(mg/d)** | **Cmax**  **(uM)** | **Reported Cardiotoxicity (reference #)** |  | **level** |
| --- | --- | --- | --- | --- | --- | --- | --- |
| Afatinib | EGFR, HER2 | EGFR^+^ NSCLC | 40 | 0.052  (25.2/485.0) | N/A | N/A | low |
| Gefitinib | EGFR | NSCLC | 250 | 0.19-0.356 | N/A | N/A | low |
| Crizotinib | c-Met, ALK | ALK^+^ NSCLC | 500 | 0.22-0.3 | Sinus bradycardia, QT prolongation^34-36^ | QT prolongation: 2.1%-5%, 90.4% bradycardia^1^; | mid |
| Dasatinib | Abl, Src, c-Kit | Ph^+^ CML,  Ph^+^ ALL | 100 | - | N/A; cardiac ischemic; cardiac-related fluid retention; QT Prolongation; peripheral arterial occlusive^32^ | Myocardial ischemia / infarction: 3.9%;  Congestive heart failure: 1.62%;  QT prolongation: 7% (arrhythmia and palpitations); | mid |
| Nilotinib | Bcr-Abl | Ph^+^ CML, GIST | 800 | 3.78^33^ | QT prolongation; vascularo cclusive events; hypertension; ischemic cerebrovascular event; peripheral artery disease^30,31^ | Hypertension: 4-10.4%; Myocardial ischemia / infarction: 7.5%-13.4%^7^; QT prolongation: 11%, 20% electrocardiographic changes^29^; Thromboembolism: 15.2% | mid |
| Ponatinib | VEGFR2, PDGFR, FGFR1, Abl, Src | CML, Ph^+^ ALL | 45 | 0.09-0.155 | severe narrowing of vessels; heart failure; left ventricular dysfunction; hypertension | Hypertension: 32%, 67%, 25% in CML patient; Myocardial ischemia / infarction: 4%-28%; Congestive heart failure: 1-9%; QT prolongation: 2%; arrhythmias: 19%;  Thromboembolism : 35%, 5% | high |
| Sorafenib | VEGFR2, PDGFR, Raf | HCC, RCC, thyroid, GIST | 800 | 6.17 | Hypertension; QT prolongation; LVEF decrease; congestive heart failure; myocardial infarction^28^ | Hypertension: 9.4% (HCC), 16.9% (RCC), 40.6% (DTC); Myocardial ischemia / infarction:2.7% (HCC), 2.9% (RCC) , 1.9% (DTC), 2-3% (NSCLC); Congestive heart failure: 1.9%;^2, 27, 28^ | high |
| Sunitinib | VEGFR2, PDGFR, c-Kit | GIST, PNET, RCC | 50 | - | Hypertension; CHF; LVEF decrease; myocardial infarction; QT prolongation ^4, 26^ | Hypertension: 15% (GIST), 34% (RCC), 27% (pNET), 47%, 23%;  Myocardial ischemia /infarction: 1.22%;  Congestive heart failure: 3% (RCC, pNET), 8%;  Left ventricular ejection fraction decrease: 11% (GIST) , 27% (RCC), 10%, 20%;  QT prolongation: 20%^4, 26^ | high |

**Table S1** **Literature review on TKI cardiotoxicity**

**Table S2 Differentially expressed genes of ER stress in cardiomyocytes treated with sorafenib**

| Gene name |  | Sorafenib 3.16 µM 168 hours | | Sorafenib 10 µM 24 hours | |
| --- | --- | --- | --- | --- | --- |
|  |  | logFC | FDR | logFC | FDR |
| *ATF4* |  | 2.09 | 7.30E-17 | 1.91 | 1.44E-21 |
| *CHAC1* |  | 3.62 | 6.88E-14 | 4.55 | 3.78E-30 |
| *DDIT3* |  | 4.28 | 1.87E-37 | 4.73 | 3.10E-66 |
| *TRIB3* |  | 5.51 | 2.19E-28 | 5.56 | 5.04E-43 |
| *XBP1* |  | 1.42 | 8.34E-16 | 1.79 | 3.13E-36 |
| *DNAJB9* |  | 1 | 8.43E-11 | 1.43 | 2.96E-31 |
| *ATF6* |  | 0.64 | 3.31E-09 | 0.91 | 1.05E-26 |

**Table S3 ARRIVE checklist**


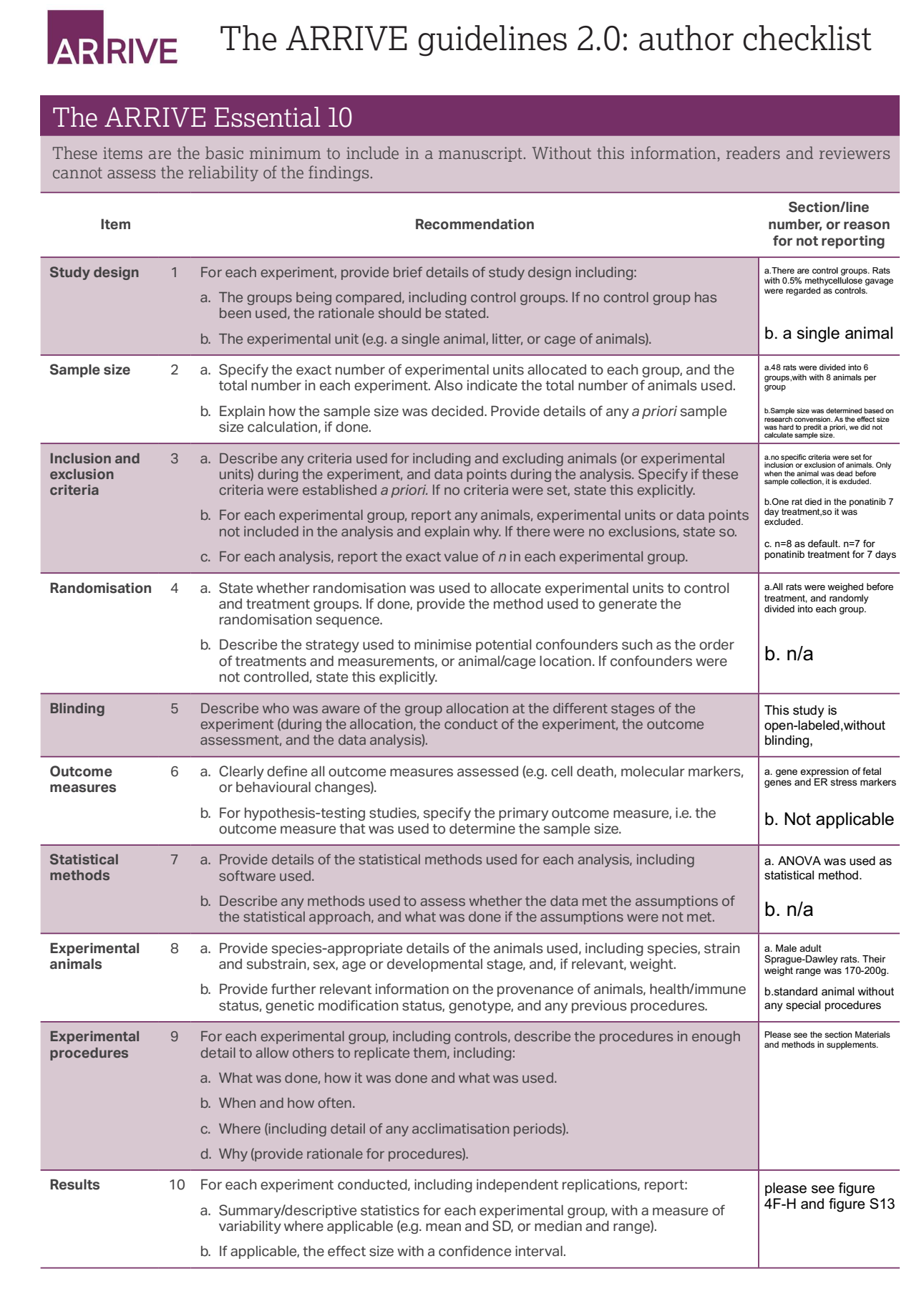

Supplement: Supplementary file 1 — Additional file 1: Table S1. An introduction to the pharmacology and toxicology of TKI drugs of different cardiotoxicity levels. Table S2. Endoplasmic reticulum stress gene markers under different conditions of sorafenib. Table S3. The ARRIVE checklist. [file 12916_2023_2838_MOESM1_ESM.docx]
