# Supplementary material for: Three tyrosine kinase inhibitors cause cardiotoxicity by inducing endoplasmic reticulum stress and inflammation in cardiomyocytes
Source: BMC Med. 2023 Apr 17;21:147. doi: 10.1186/s12916-023-02838-2 (PMC10108821; doi:10.1186/s12916-023-02838-2)
Supplement: Supplementary file 2 — Additional file 2: Figure S1. EC50s of response calculated based on a four-parameter log-logistic model in the ATP fold change, related to Fig. 1. Figure S2. Seahorse experiment on acute effects of TKIs on mitochondrial oxygen consumption and extracellular acidification, related to Fig. 1. Figure S3. Mitochondrial membrane potential changes in response to TKIs observed by TMRE staining and its fold change, related to Fig. 1. Figure S4. Clustering of TKI-induced transcriptome data based on tSNE analysis, related to Fig. 2. Figure S5. Cluster 0, 2, 3, 4, 6 contained over 10 significant DEGs found by log2-based fold changes, related to Fig. 2. Figure S6. Expression of genes related to tRNA aminoacylation for protein translation in different clusters or in response to different drugs, related to Fig. 2. Figure S7. Good quality and consistency of 3’DGE-UMI RNA-seq, related to Fig. 2. Figure S8. The Jackstraw plot of the top 15 principal components in the tSNE analysis, related to Fig. 2. Figure S9. The number of unique genes, total counts, and proportion of mitochondrial DNA present in the 3'DGE-UMI RNA-seq data, related to Fig. 2. Figure S10. Correlation analysis between mitochondrial DNA and total counts or between unique genes and total counts in 3’DGE-UMI RNA-seq data, related to Fig. 2. Figure S11. Comparison of differentially expressed genes detected by 3'DGE-UMI and bulk RNA-seq for sorafenib and sunitinib treatments, related to Fig. 2. [file 12916_2023_2838_MOESM2_ESM.docx]

**Three tyrosine kinase inhibitors cause cardiotoxicity by inducing endoplasmic reticulum stress and inflammation**

Huan Wang^1,#,*^, Yiming Wang^1,#^, Jiongyuan Li^1^, Ziyi He^1^, Sarah A. Boswell^2^, Mirra Chung^2^, Fuping You^1^, Han Sen^3^

1. Institute of Systems Biomedicine, School of Basic Medical Sciences, Peking University Health Science Center, Beijing, 100191, China

2. Laboratory of Systems Pharmacology, Department of Systems Biology, Harvard Medical School, Boston, Massachusetts 02115, USA

3.Key Laboratory of Carcinogenesis and Translational Research (Ministry of Education), Peking University Cancer Hospital & Institute, Beijing 100142, China.

* Corresponding author, email: [huan_sharon_wang@pku.edu.cn](mailto:huan_sharon_wang@pku.edu.cn)

# these authors contributed equally


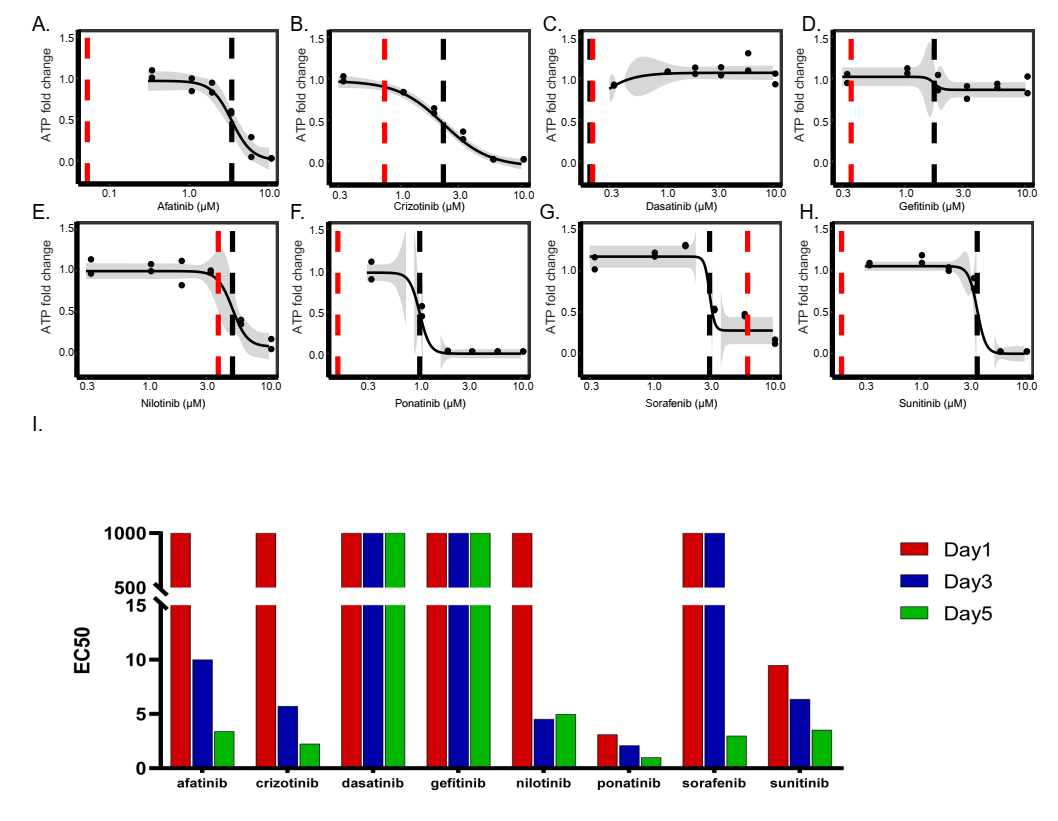


**Fig S1 EC50 of dose responses in the ATP fold change in Figure 1.** (A-H) Dose response curves based on ATP fold changes to TKIs at day 5 were fitted by a four-parameter log-logistic model. EC50 and Cmax for each drug were plotted as black or red dashed lines. Gray shade represents 95% confidence interval of responses. (I) For dose-response curves of ATP fold changes from day 1 to day 5, EC50s were calculated based on a four-parameter log-logistic model and plotted as grouped by drugs. EC50s of most drugs, except dasatinib and gefitinib, were decreased over time.


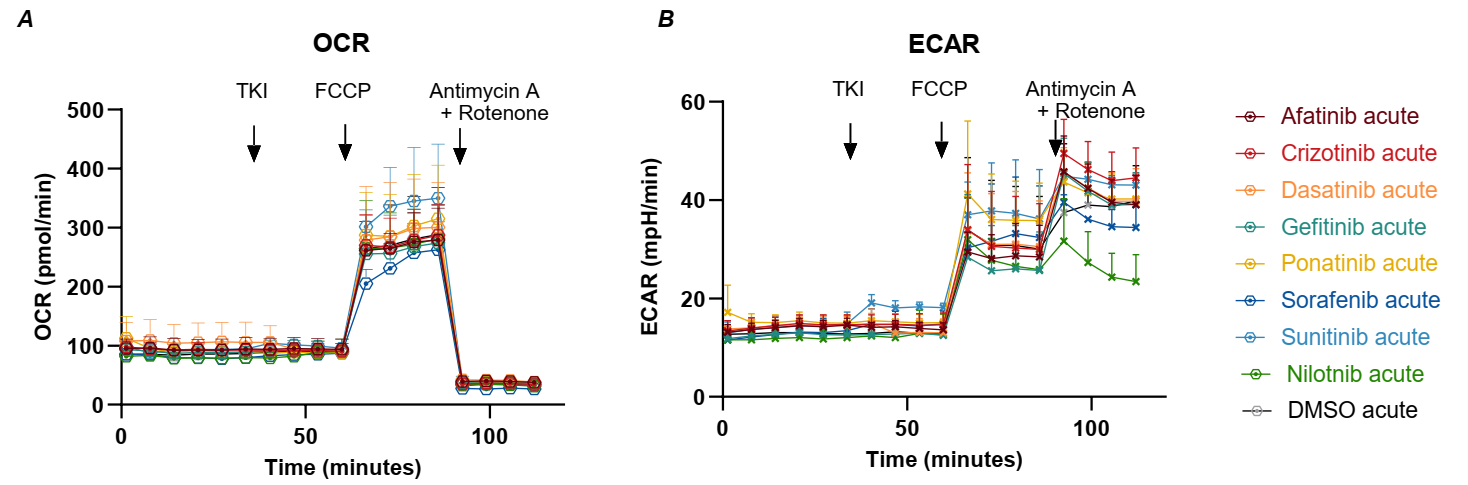


**Fig S2 Acute effects of TKIs on mitochondrial oxygen consumption and extracellular acidification.** Seahorse experiment was done to measure the effect of TKIs on mitochondrial respiration and glycolysis. TKIs were added acutely and the measurements were done a few minutes after TKI addition. (A) Oxygen consumption rate of different treatments (B) Extracellular acidification rate measured for different treatments.


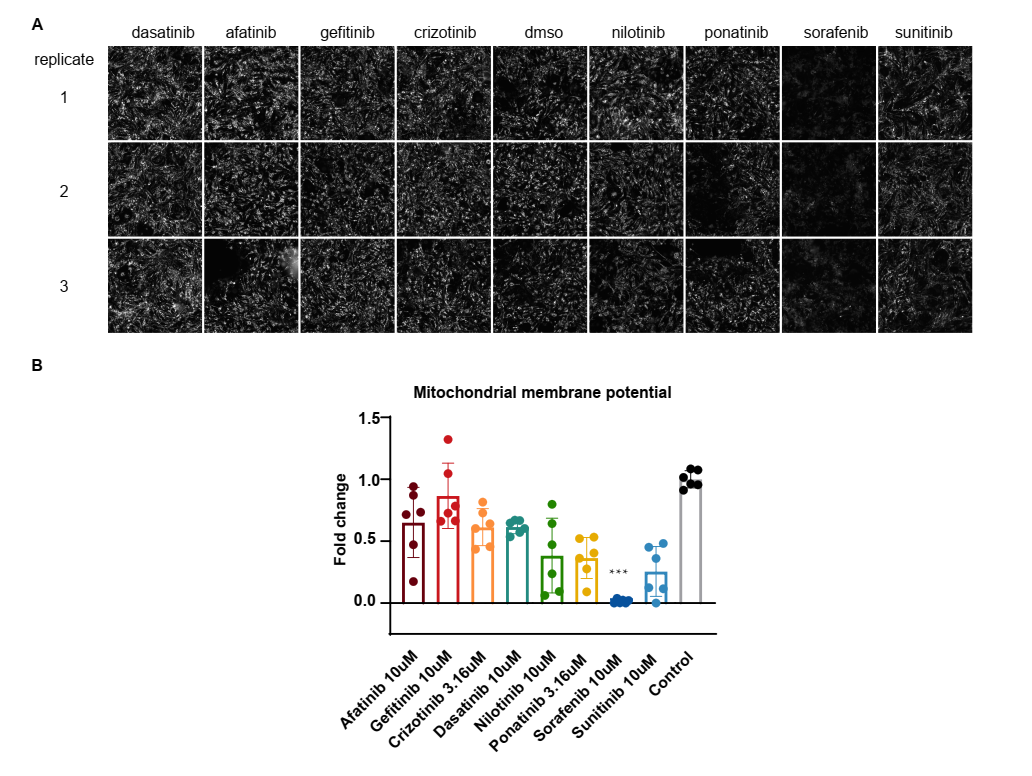
**Fig S3 Mitochondrial membrane potential changes in response to TKIs.** (A) Raw images of TMRE staining in NRCMs treated with different drugs. (B) Fold changes in mitochondrial membrane potential at different treatments.


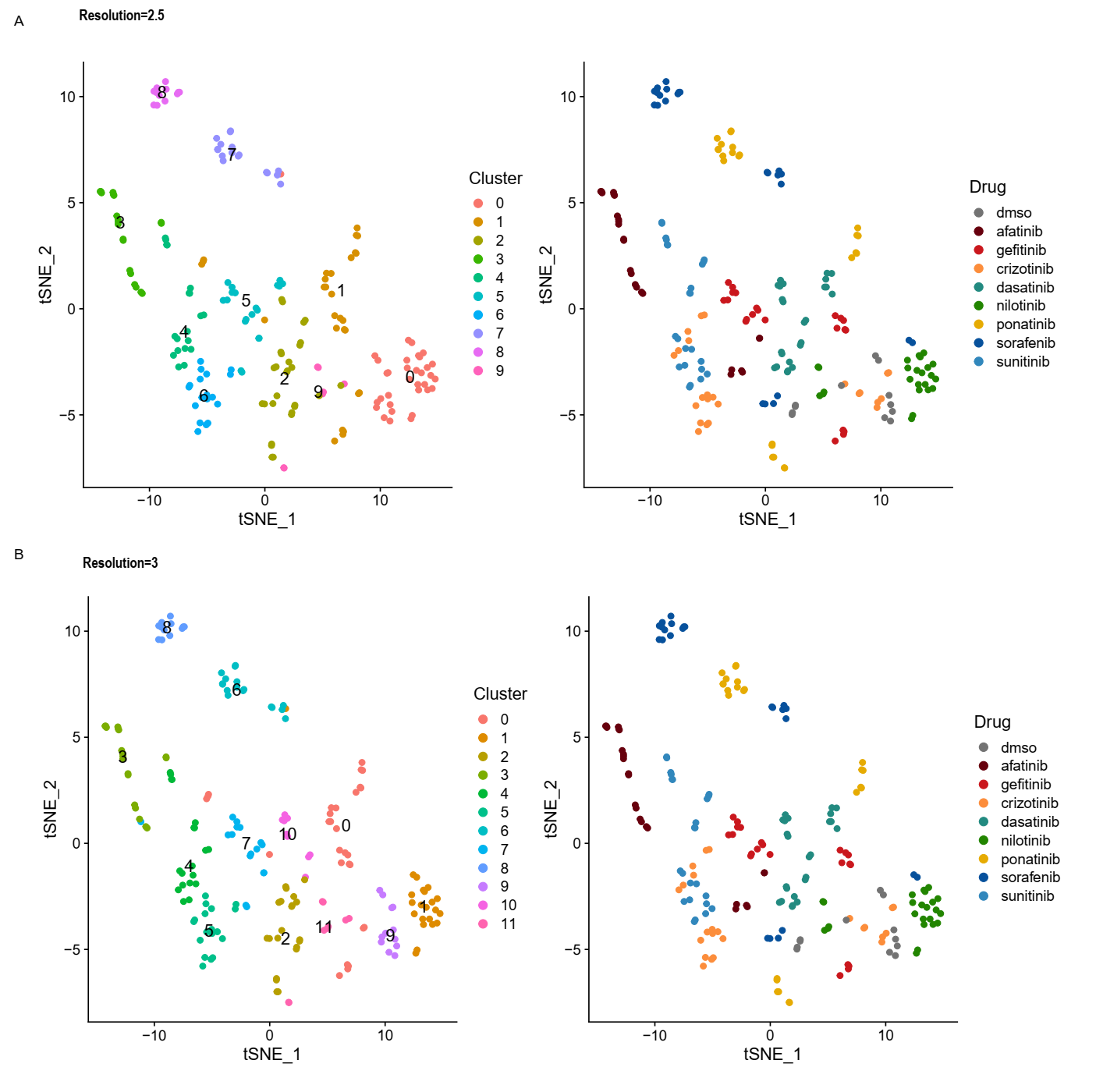


**Figure S4 Clustering of transcriptome data based on tSNE analysis.** (A) Clustering of TKI-induced transcriptome changes when resolution was set to be 2.5. (B) Clustering of TKI-induced transcriptome changes when resolution was set to be 3.

**
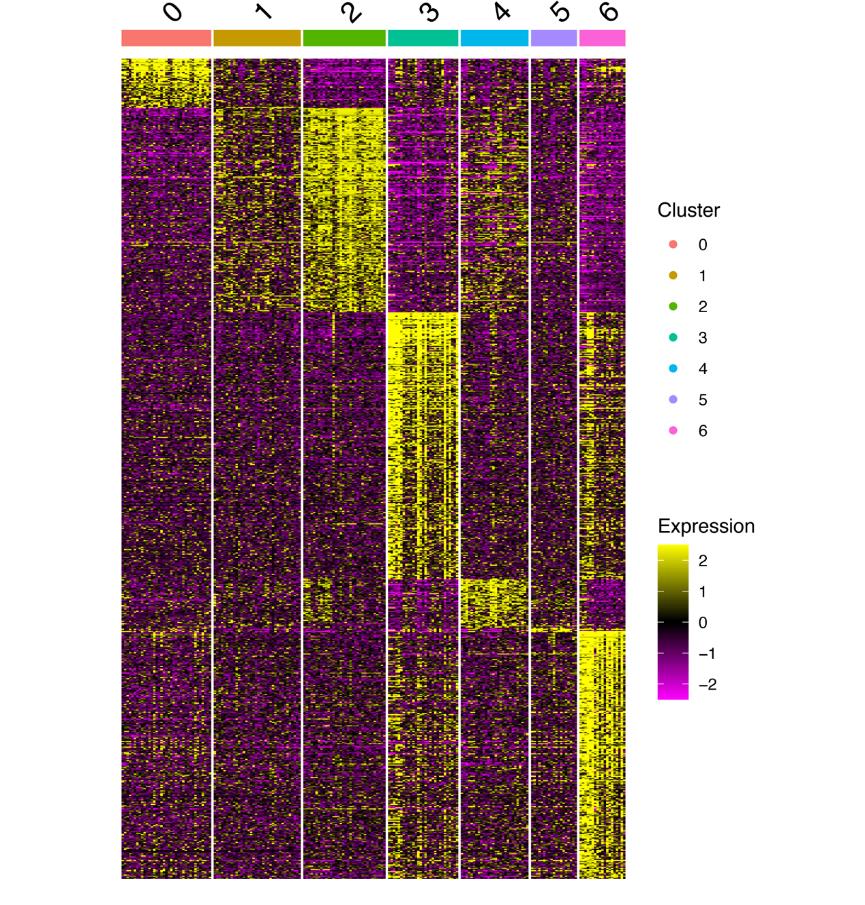
**

**Fig S5 Expression changes of significantly differentially expressed genes of each cluster** Heatmap of log2-based fold changes in gene expression for the gene markers of each cluster based on tSNE analysis.

**
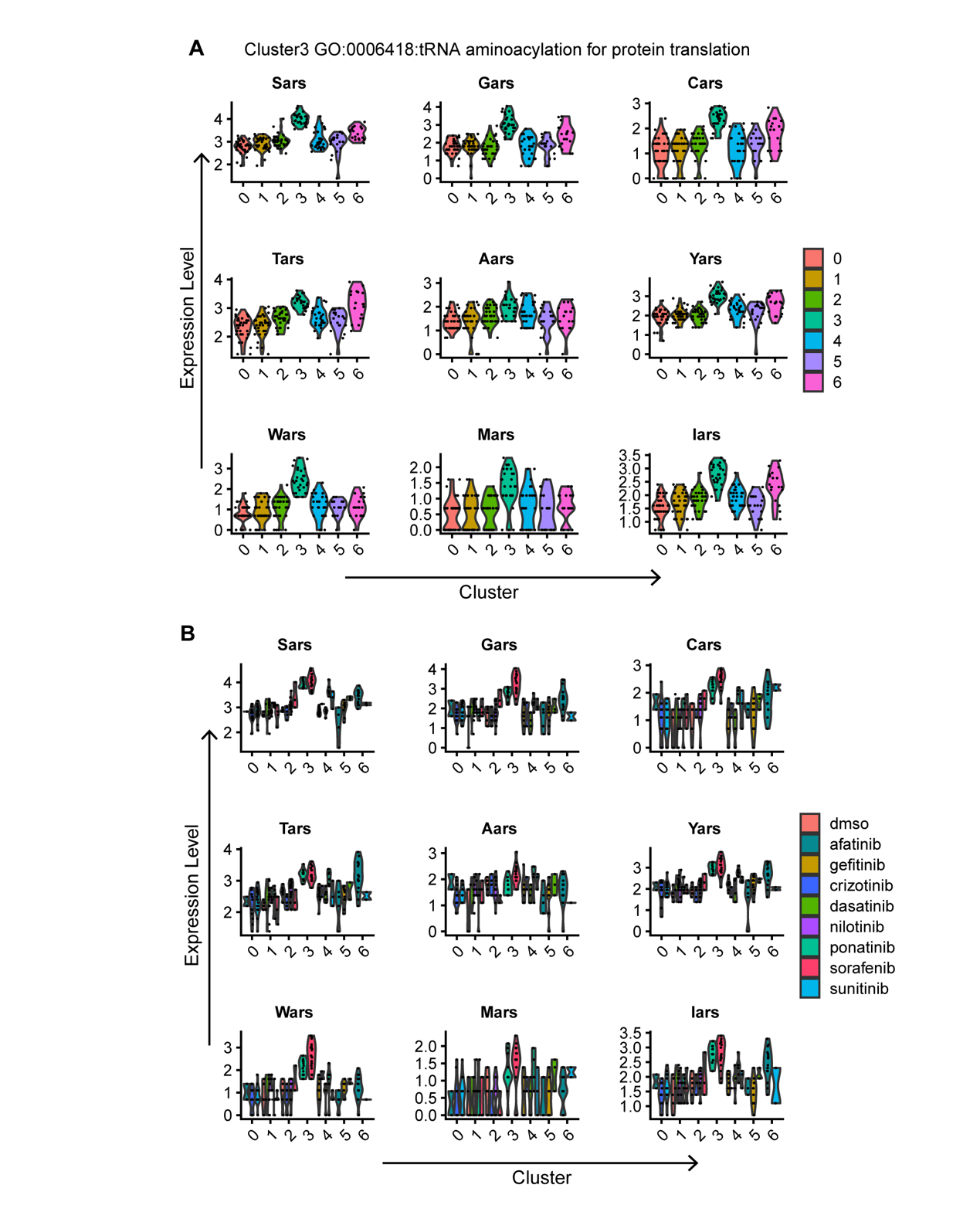
**

**Fig S6 Expression of genes related to tRNA aminoacylation for protein translation** (A) Expression levels of *Sars*, *Gars*, *Cars*, *Tars*, *Aars*, *Yars*, *Wars*, *Mars* and *Iars*, which are representative genes for the GO term of tRNA aminoacylation for protein translation, were increased in Cluster 3. (B) Expression levels of the same genes as in (A) grouped by clusters and drugs. Both sorafenib and ponatinib upregulated these genes.

**
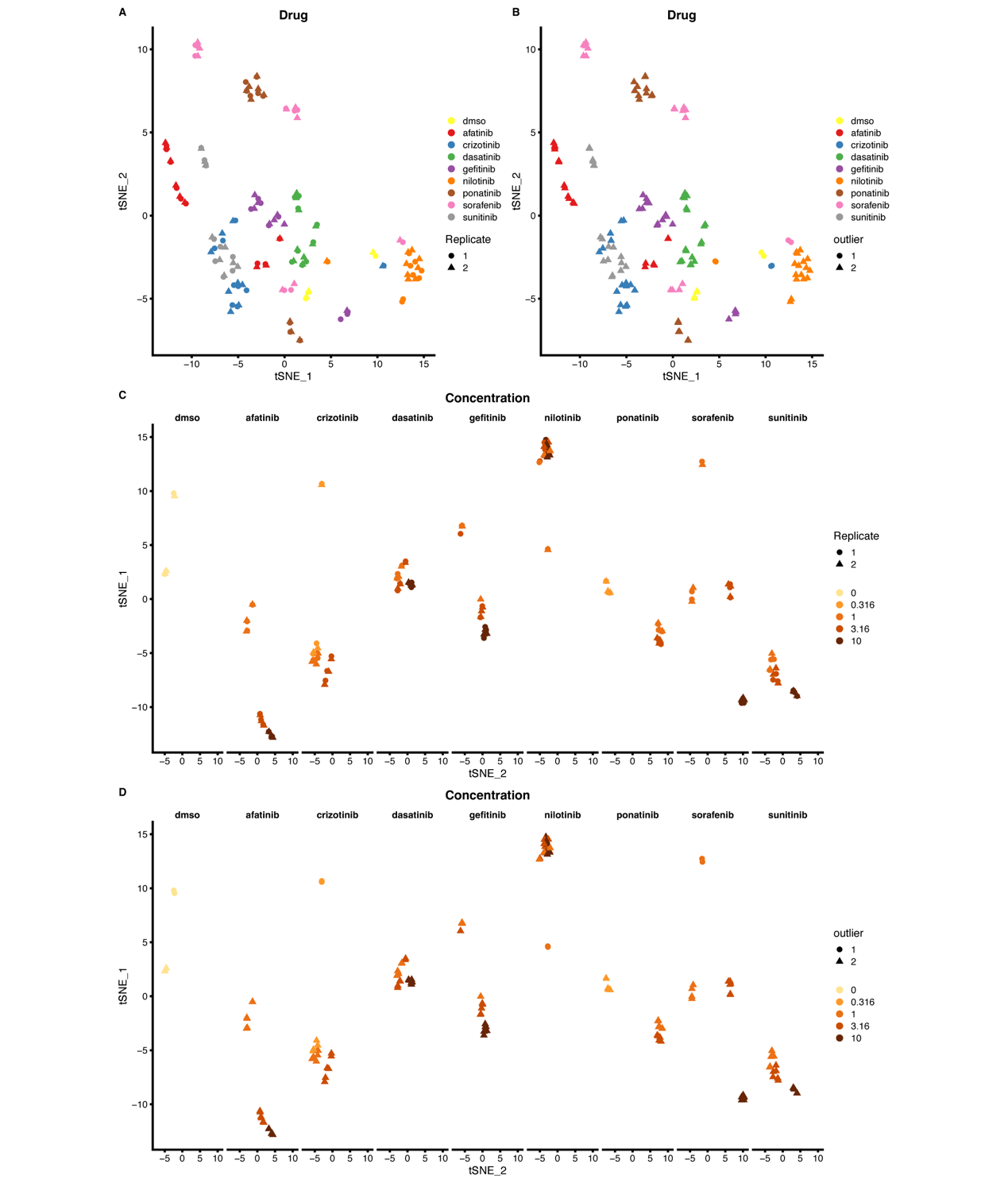
**

**Fig S7 Data quality of 3’DGE-UMI RNA-seq is good.** (A) 75 technical replicates closely mimicked their pairs as shown in the tSNE space (round dots overlapped with triangles). (B) 4 samples were potential outliers, as indicated by round dots. (C) 75 technical replicates were projected into the tSNE space but grouped by drugs. The replicates were highly similar to each other. Dots were color-coded based on concentration. (D) Potential outlier samples were projected into the tSNE space grouped by drugs. One of the dmso-, crizotinib-, nilotinib- or sorafenib-treated conditions was a potential outlier with two technical replicates (as indicated by round dots). Dots were color-coded based on concentration.


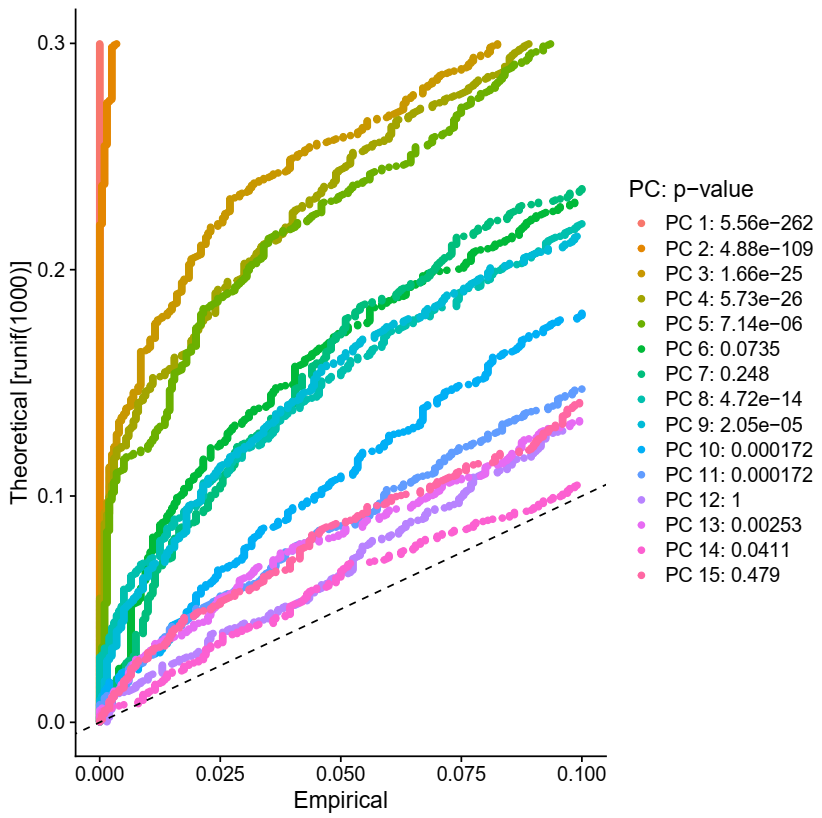


**Fig S8 The Jackstraw plot of the top 15 principal components in the tSNE analysis.** The Jackstraw analysis was used to assess the significance of principal components (PCs) use in tSNE analysis. P values of each PC were listed on the right side.

**
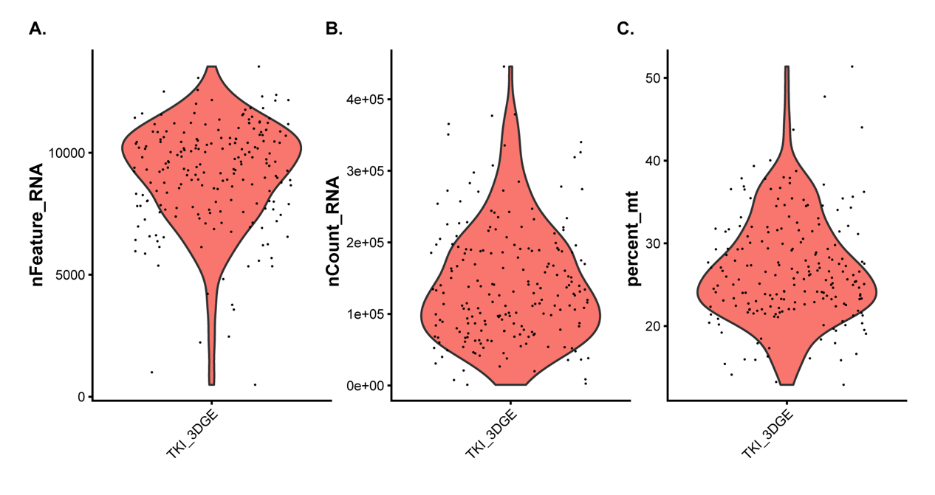
**

**Fig S9 The number of unique genes, total counts and percentage of mitochondrial DNA in the 3’DGE-UMI RNA-seq data.** (A) the number of unique genes, (B) total counts, (C) percentage ofmitochondrial DNA measured for each drug treatment in the 3’DGE-UMI RNA-seq data.

**
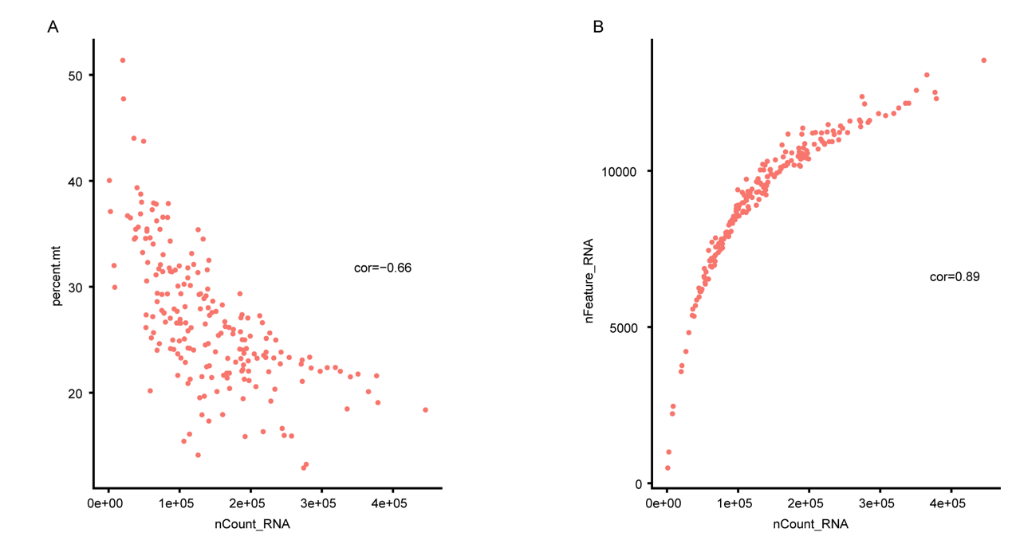
**

**Figure S10. Assessment of the 3’DGE-UMI RNA-seq data.** (A) Correlation between percentage of mitochondrial DNA and total counts of each sample was -0.66. (B) Correlation between the number of unique genes detected and total counts of each sample was 0.89.

**
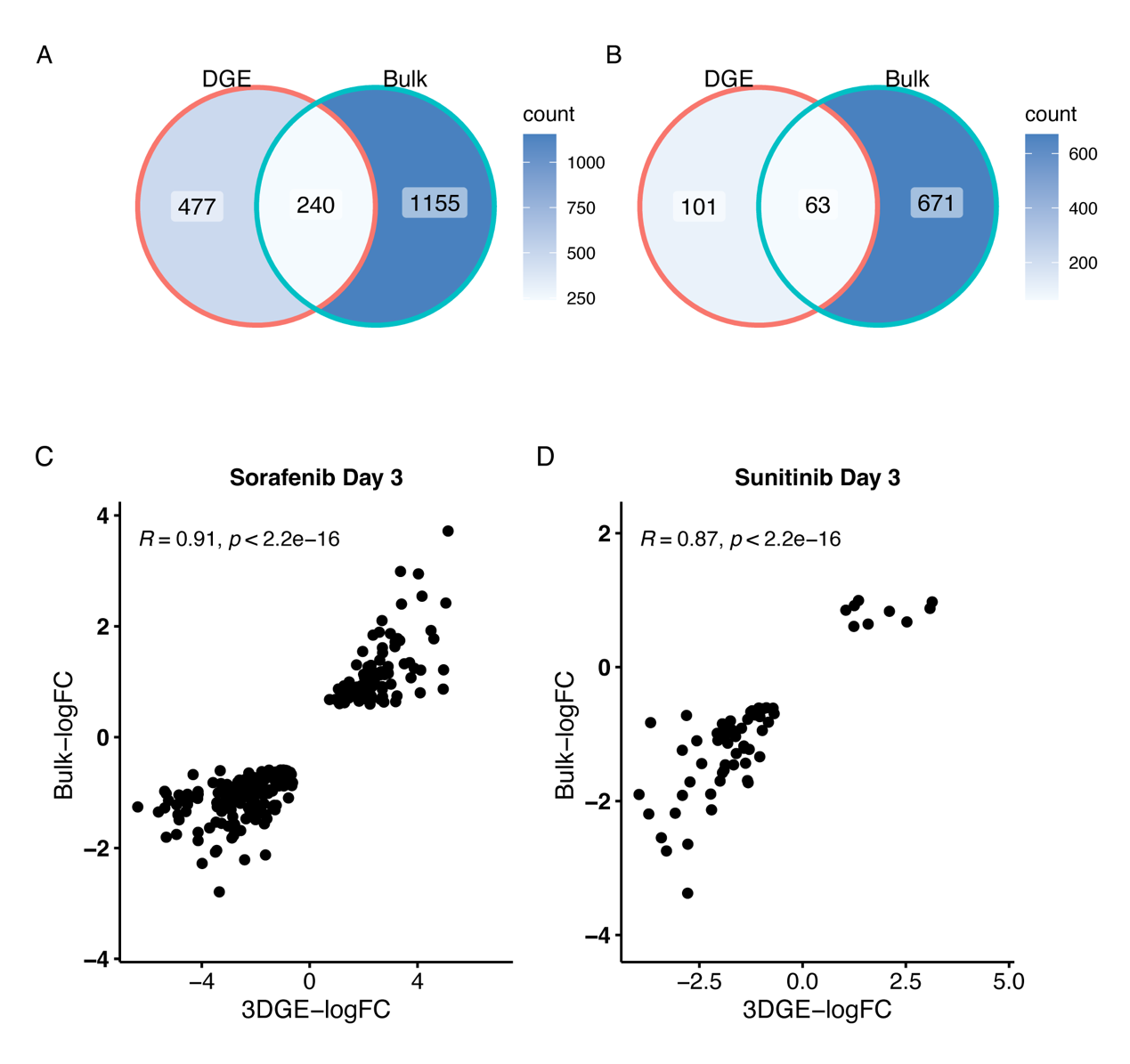
**

**Fig S11 Comparison of 3’DGE-UMI data with bulk RNA-seq data.** (A) Venn diagram of differentially expressed genes detected by 3’DGE or bulk RNA-seq for sorafenib treatment at 3.16µM and 3 days. (B) Venn diagram of differentially expressed genes detected by 3’DGE or bulk RNA-seq for sunitinib treatment at 3.16µM and 3 days. Scattered plots of log2-based fold changes of gene expression in 3’DGE and bulk RNA-seq measured for sorafenib (C) and sunitinib (D); Pearson correlation of these conditions were 0.91 and 0.87 respectively.
