# Supplementary material for: Three tyrosine kinase inhibitors cause cardiotoxicity by inducing endoplasmic reticulum stress and inflammation in cardiomyocytes
Source: BMC Med. 2023 Apr 17;21:147. doi: 10.1186/s12916-023-02838-2 (PMC10108821; doi:10.1186/s12916-023-02838-2)
Supplement: Supplementary file 3 — Additional file 3: Figure S12. Cell viability of NRCMs in response to afatinib, sorafenib, and ponatinib, related to Fig. 4. Figure S13. Low dose of TKIs induce ER stress over time in NRCMs, measured by mRNA fold changes, related to Fig. 4. Figure S14. Effects of ponatinib and sorafenib on heart weight, body weight, heart-to-body weight ratio, and Ddit3 expression in rat hearts, related to Fig. 4. Figure S15. Lipid peroxidation levels in NRCMs treated with cumene hydroperoxide and ethanol, related to Fig. 5. Figure S16. The effect of trolox on lipid peroxidation and ER stress induced by TKIs, related to Fig. 5. Figure S17. ISRIB and 4μ8c affected gene targets of Atf4 and Xbp1s induced by TKIs in NRCMs, related to Fig. 6. Figure S18. ISRIB and 4μ8c did not rescue NRCMs from cell death induced by the 3 TKIs, related to Fig. 6. Figure S19. The effects of ISRIB and 4μ8c on TKI-induced cell death examined using fluorescence imaging and quantification, related to Fig. 6. Figure S20. Persistent eIF2α phosphorylation up-regulated Nfkb1 and Il6 expression induced by 3 TKIs in H9C2 cells, but not Il1b or Tnf, related to Fig. 6. [file 12916_2023_2838_MOESM3_ESM.docx]

**Three tyrosine kinase inhibitors cause cardiotoxicity by inducing endoplasmic reticulum stress and inflammation**

Huan Wang^1,#,*^, Yiming Wang^1,#^, Jiongyuan Li^1^, Ziyi He^1^, Sarah A. Boswell^2^, Mirra Chung^2^, Fuping You^1^, Han Sen^3^

1. Institute of Systems Biomedicine, School of Basic Medical Sciences, Peking University Health Science Center, Beijing, 100191, China

2. Laboratory of Systems Pharmacology, Department of Systems Biology, Harvard Medical School, Boston, Massachusetts 02115, USA

3.Key Laboratory of Carcinogenesis and Translational Research (Ministry of Education), Peking University Cancer Hospital & Institute, Beijing 100142, China.

* Corresponding author, email: [huan_sharon_wang@pku.edu.cn](mailto:huan_sharon_wang@pku.edu.cn)

# these authors contributed equally


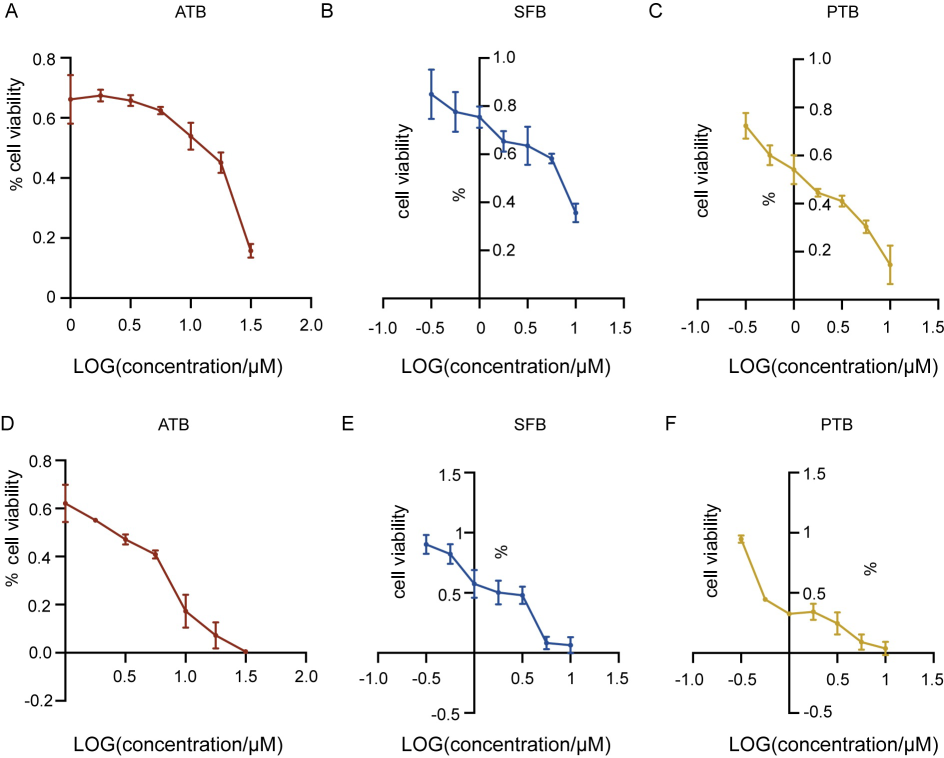


**Fig S12** **Cell viability of NRCMs in response to the 3 TKIs.** (A-C) NRCMs viability measured by CCK8 in response to various doses of afatinib, sorafenib or ponatinib for 24-hr. (D-F) NRCMs viability measured by CCK8 in response to various doses of afatinib, sorafenib or ponatinib for 72-hr. Data were presented as mean ± SEM (n=3).

**
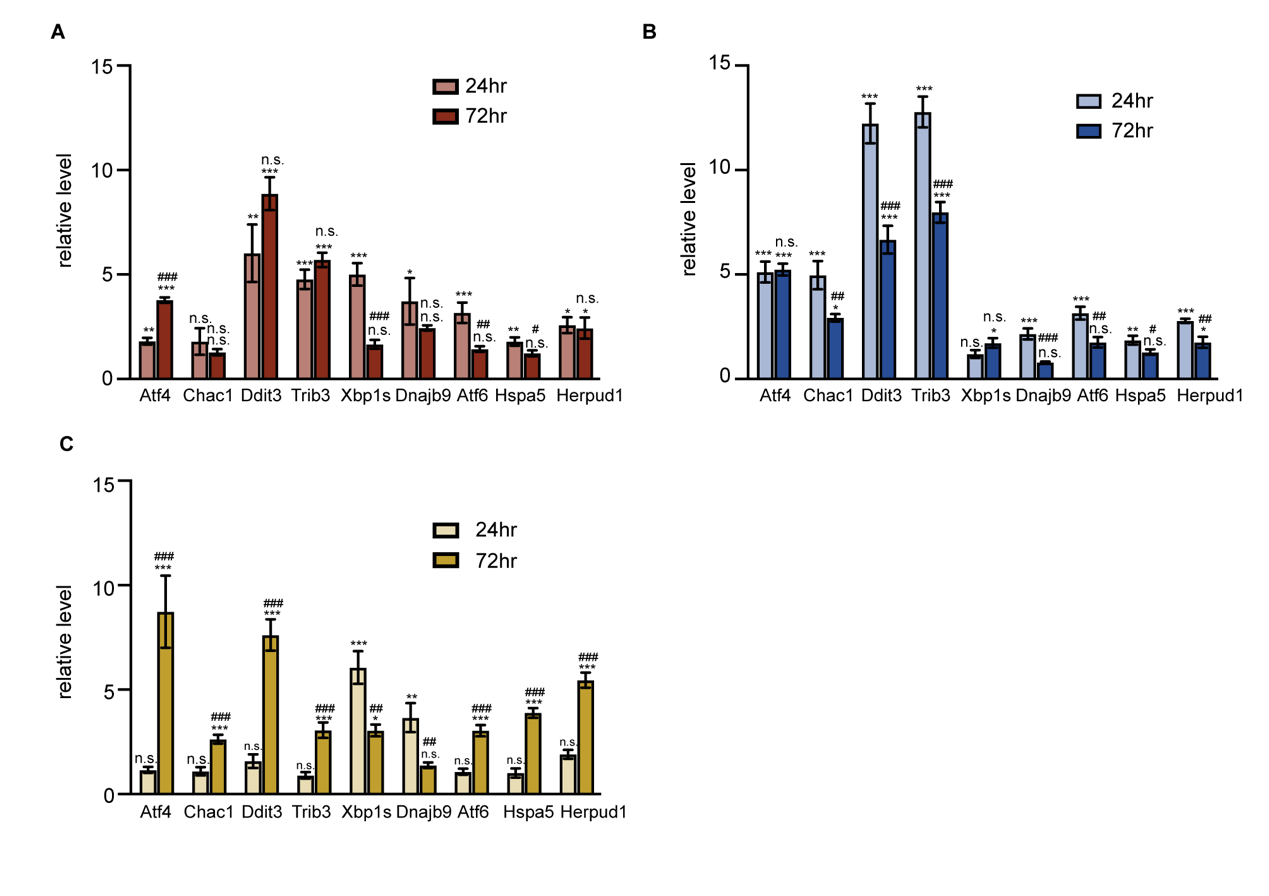
**

**Fig S13 Low dose of afatinib, sorafenib and ponatinib induce ER stress over time in NRCMs.** (A-C) Fold changes of Atf4, Chac1, Ddit3, Trib3, Xbp1s, Dnajb9, Atf6, Hspa5 and Herpud1 mRNAs in NRCMs treated with 5.62μM afatinib, 3.16μM sorafenib, or 1.78μM ponatinib for 24 or 72 hours. Data were presented as mean ± SEM (n=3) and analyzed using ANOVA analysis. Bottom asterisks denote significance between each group with the DMSO vehicle control group, *p < 0.05, **p < 0.01, ***p < 0.001. Top hashes denote significance between the longer versus the shorter time point, ^#^p < 0.05, ^##^p < 0.01, ^###^p < 0.001.


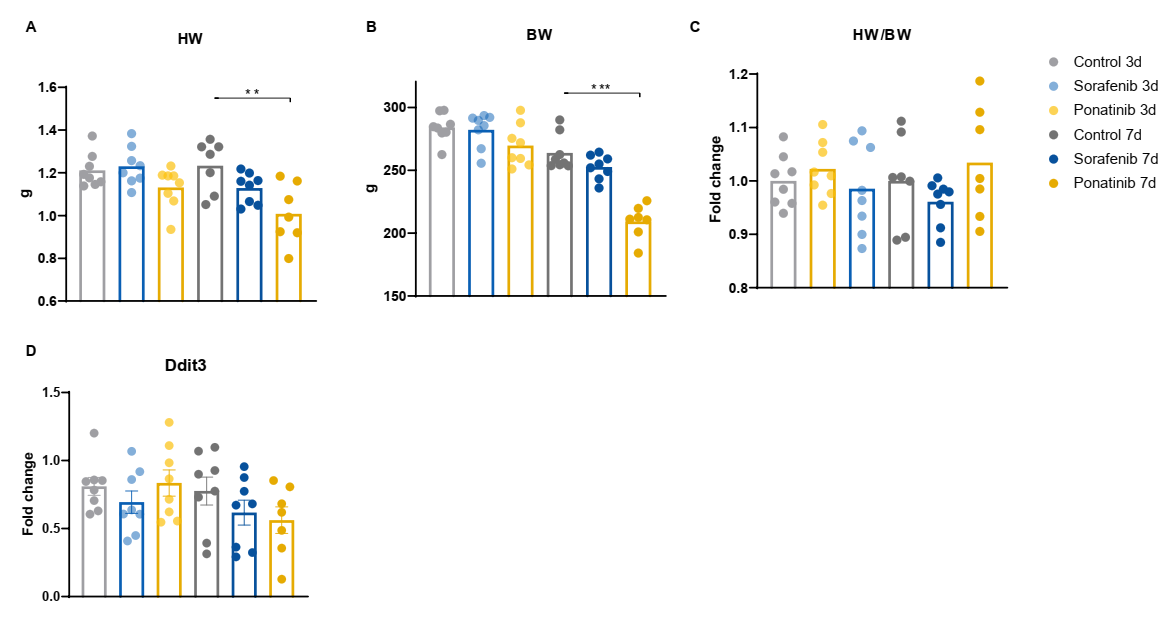


**Fig S14 Heart weight, body weight and gene expression of drug-treated rats.** Sprague-Dawley rats were gavaged with either ponatinib (15mg/kg) or sorafenib (50mg/kg) once daily. (A) Heart weights of animals at the time of sample collection. (B) Body weights of animals at the time of sample collection (older rats were used for the 3-day gavage experiment, that’s why day 3 animals were heavier than the day 7 animals) (C) Fold changes in the heart-to-body weight ratio of animals at the time of sample collection. (D) Ddit3 expression of TKI-treated rat hearts.


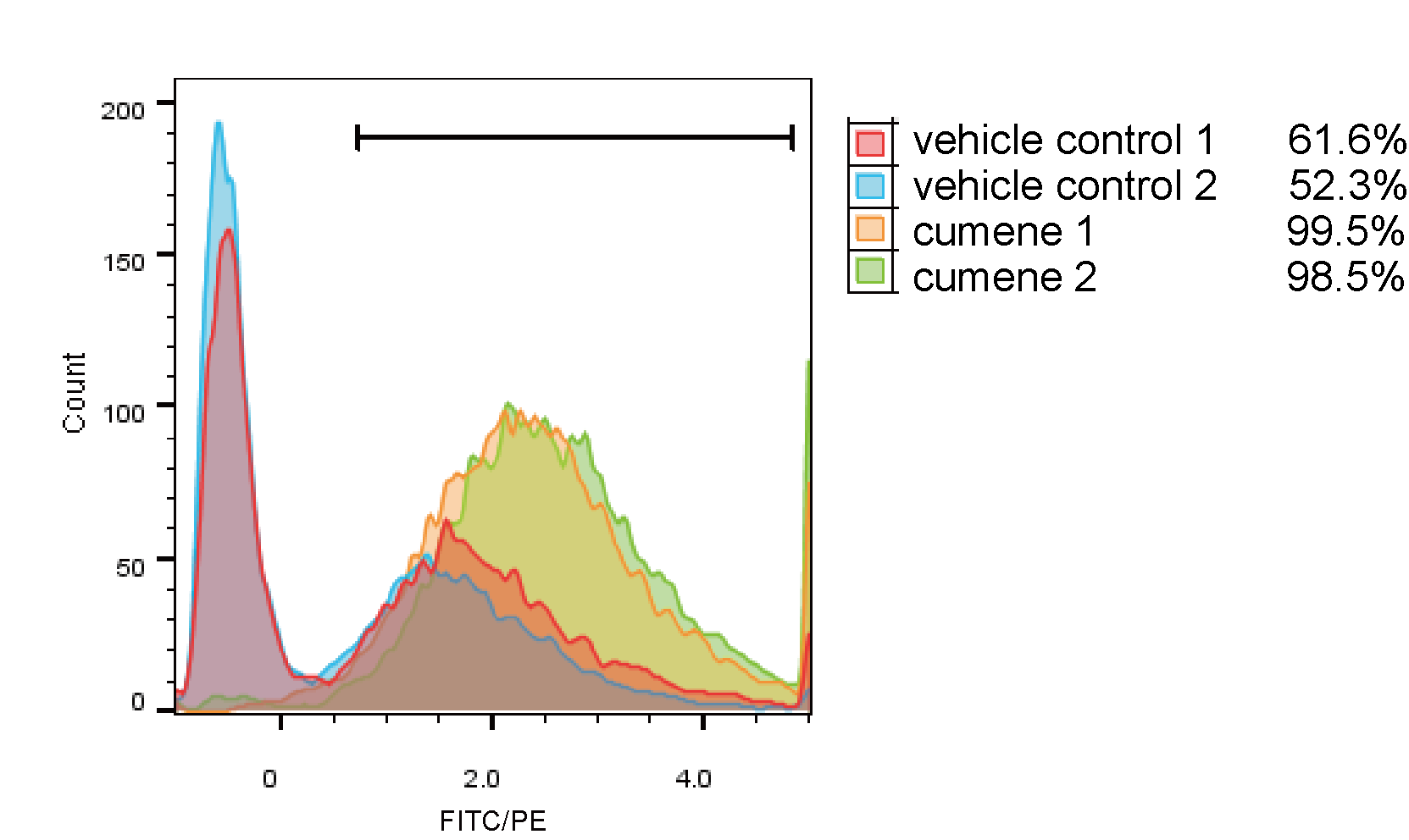


**Fig S15 Lipid peroxidation measured by flow cytometry.** NRCMs were treated with ethanol (vehicle control) or cumene hydroperoxide (100 µM) for 3 hours. The ratio of FITC/PE was derived from flow cytometry measurement and plotted as histograms. The percent of cells in the gate was listed for each condition on the right.


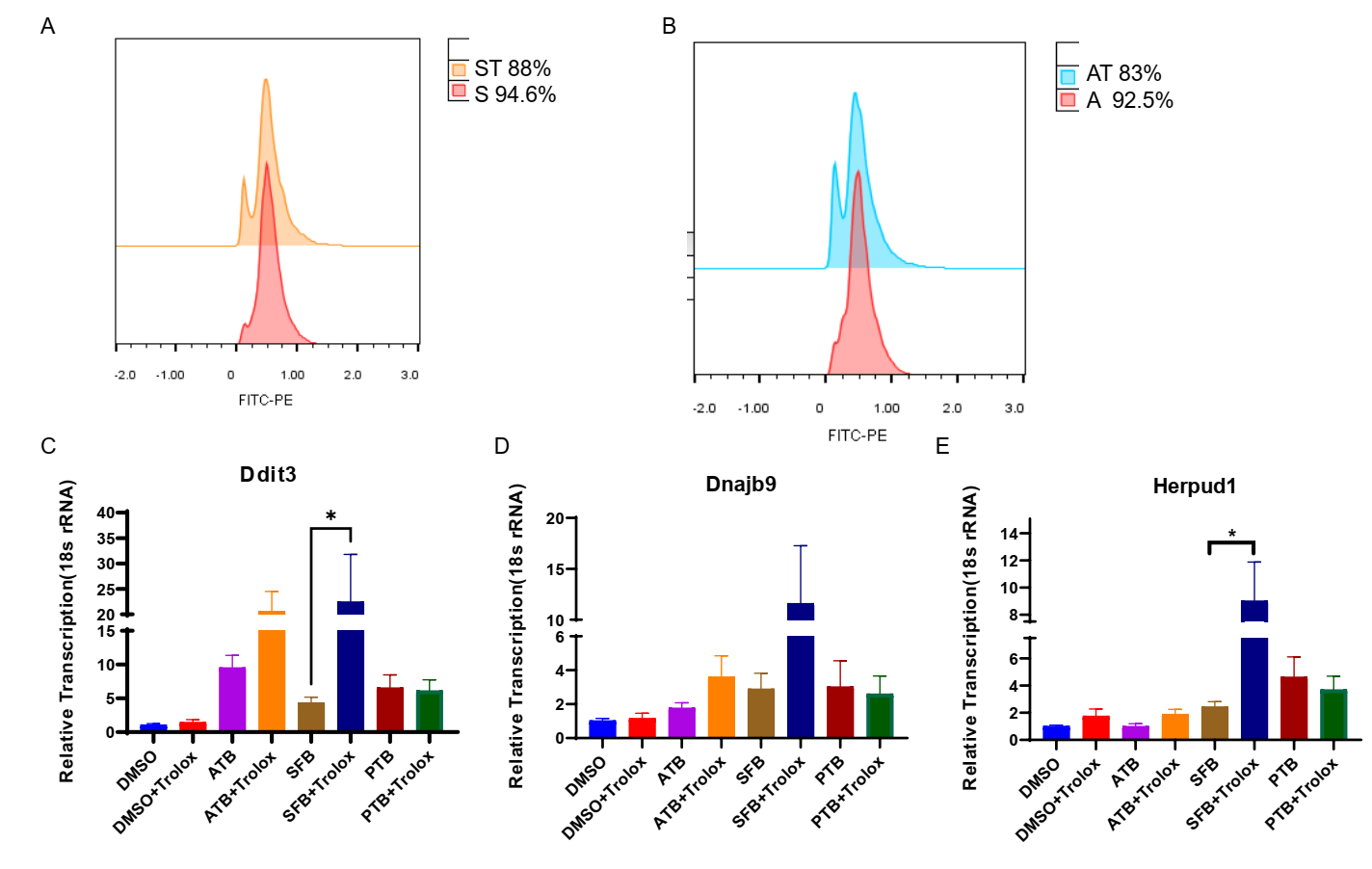


**Fig S16 Effects of trolox, an anti-oxidant, on ER stress induced by three TKIs.** (A) Percent of cells with high lipid peroxidation induced by S (sorafenib 10 µM) or ST (sorafenib 10 µM with trolox 1 mM) as measured by flow cytometry. (B) Percent of cells with high lipid peroxidation induced by A (afatinib 10 µM) or AT (afatinib 10 µM with trolox 1 mM) as measured by flow cytometry. (C-E) Expression of Ddit3, Dnajb9 and Herpud1 in NRCMs treated with the vehicle control (DMSO) or with TKIs (+trolox). *: p<0.05.

**
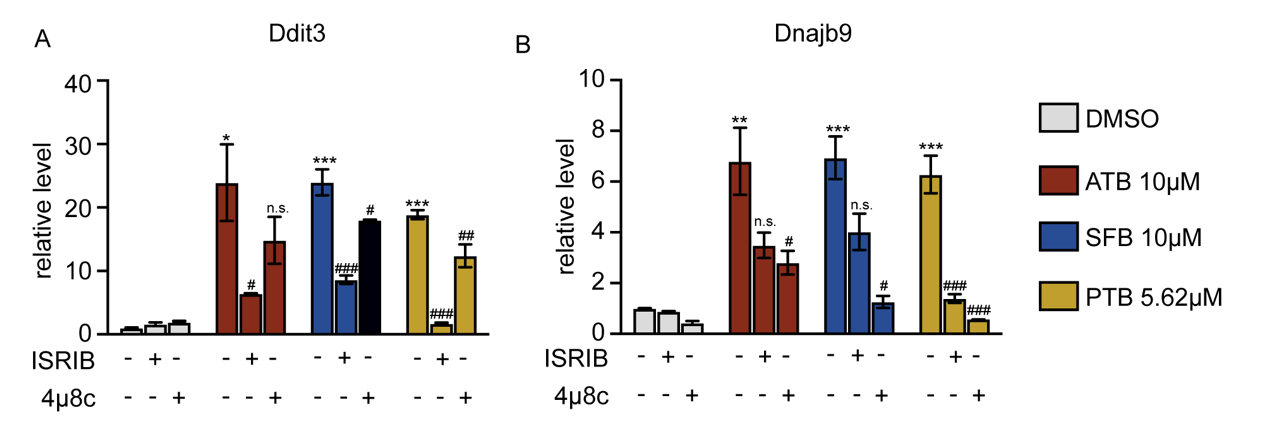
**

**Fig S17 Effects of ISRIB and 4μ8c on gene targets of Atf4 and Xbp1s** (A-B) Fold changes of Ddit3 (or CHOP) and Dnajb9 mRNAs in NRCMs treated with 3 TKIs (10μM afatinib, 10μM sorafenib, 5.62μM ponatinib) in combination with or without ISRIB (200nM) or 4μ8c (10μM) for 24-hr. Data were presented as mean ± SEM (n=3) and analyzed using ANOVA analysis. *p < 0.05, **p < 0.01, ***p < 0.001 versus the DMSO vehicle control group. ^#^p < 0.05, ^##^p < 0.01, ^###^p < 0.001 versus the TKI treatment alone.


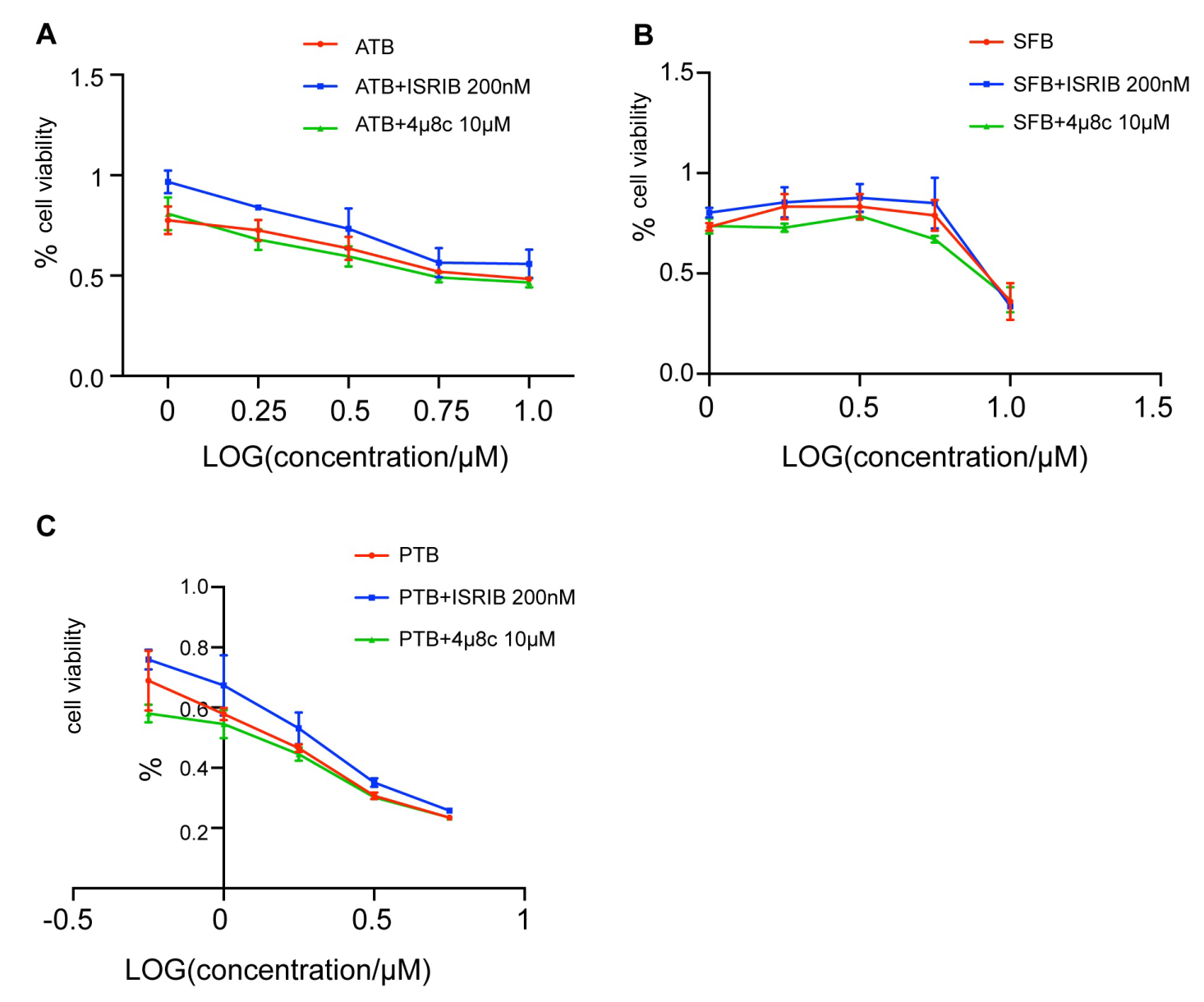


**Fig S18 ISRIB or 4μ8c does not rescue cell death induced by the 3 TKIs in NRCMs** (A-C) NRCMs viability measured by CCK8 in response to various doses of afatinib, sorafenib or ponatinib in combination with or without ISRIB (200nM) or 4μ8c (10μM) for 24-hr. Data were presented as mean ± SEM (n=3).

**
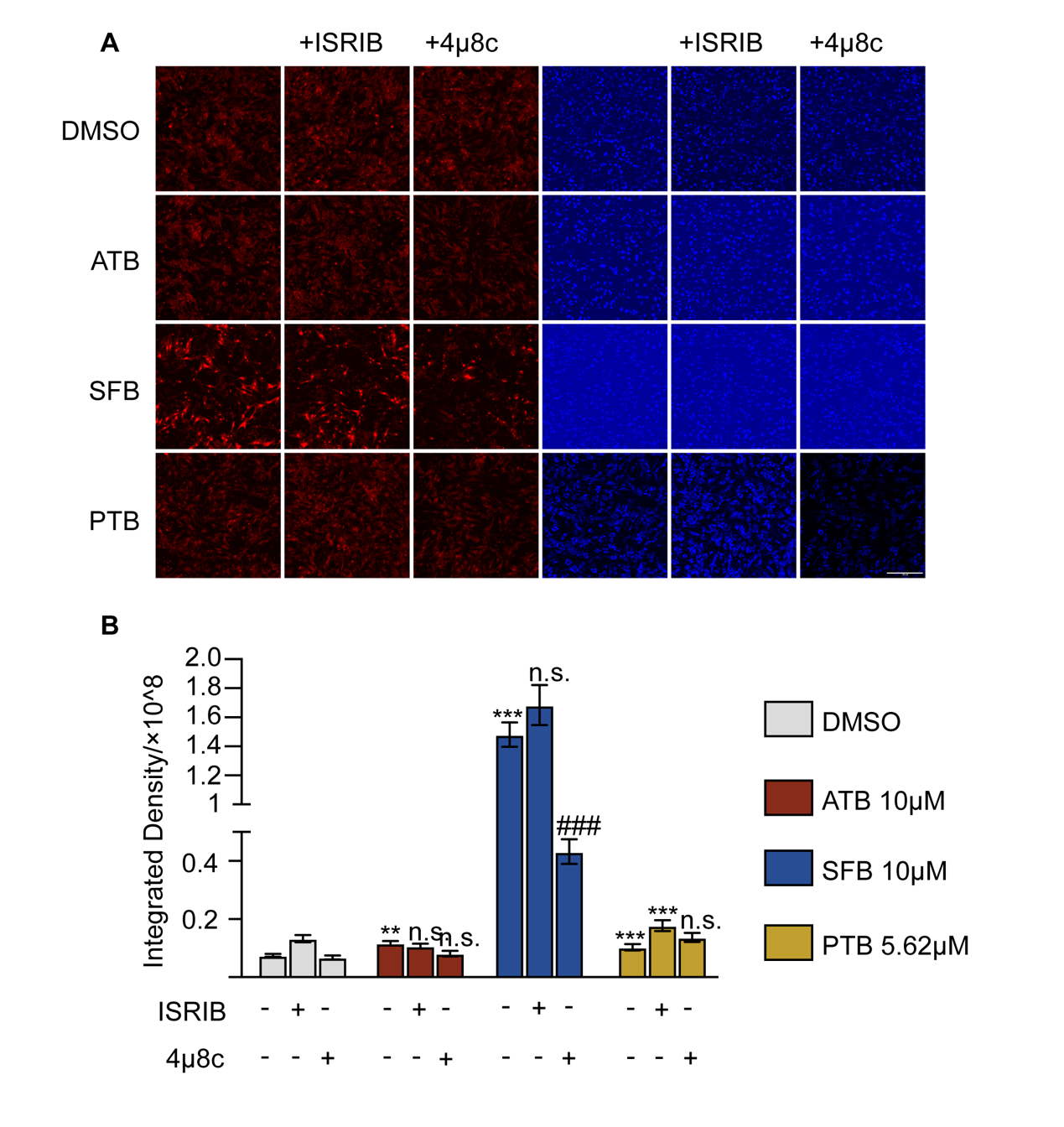
**

**Fig S19 ISRIB or 4μ8c has different effects on cell death caused by three TKIs.** (A) Representative fluorescence images of NRCMs under the treatment of the 3 TKIs (10μM afatinib, 10μM sorafenib or 5.62μM ponatinib) in combination with or without ISRIB (200nM) or 4μ8c (10μM) for 24-hr and stained with PI (red) and Hoechst33342 (blue). (B) Quantification of PI fluorescence intensity of (A). Data were presented as mean ± SEM (n=3) and analyzed using ANOVA analysis. *p < 0.05, **p < 0.01, ***p < 0.001 versus the DMSO vehicle control group. ^#^p < 0.05, ^##^p < 0.01, ^###^p < 0.001 versus the TKI treatment alone.

**
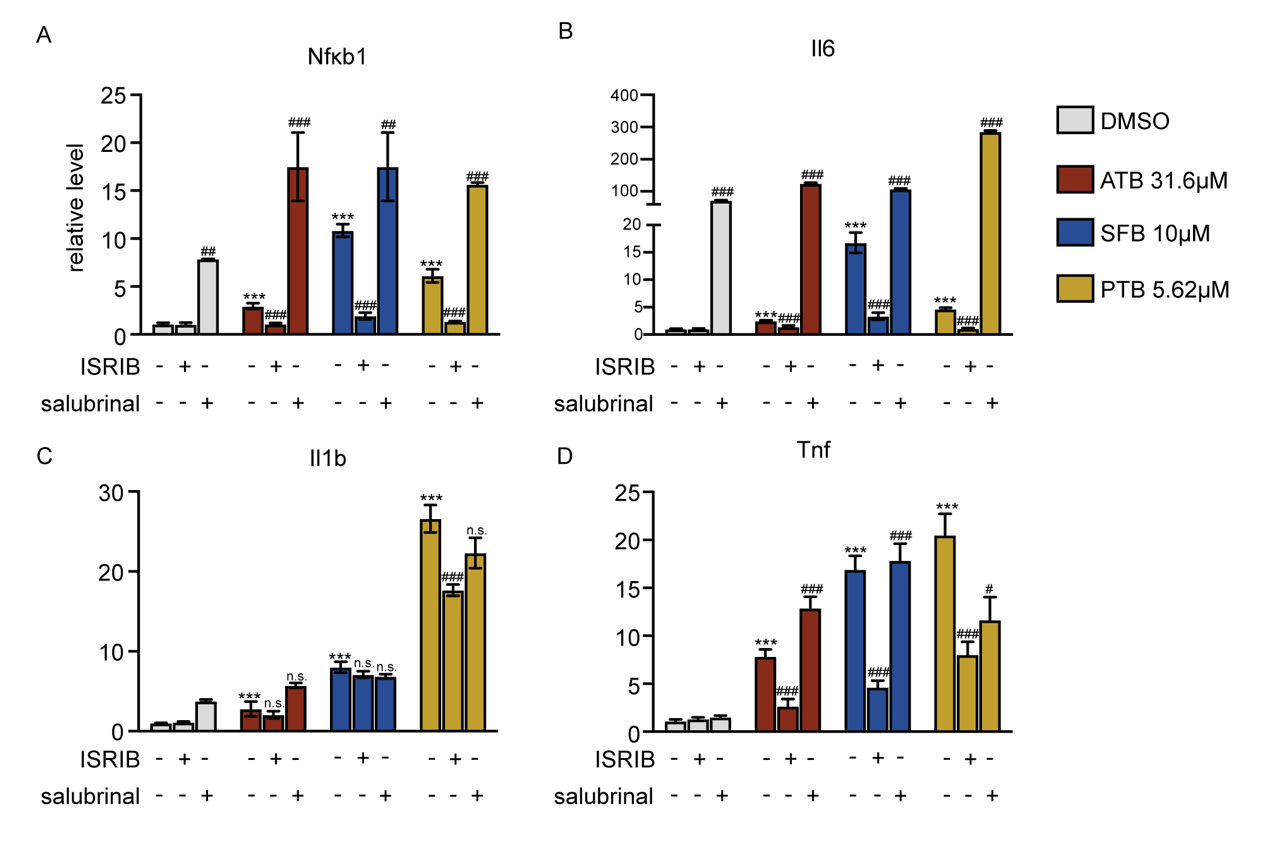
**

**Fig S20** **Persistence of eIF2α phosphorylation determines the up-regulation of Nfkb1 and Il6, but not Il1b or Tnf, by the 3 TKIs.** (A-D) Fold changes of Nfkb1, Il6, Il1b and Tnf mRNAs in H9C2 cells treated with 3 TKIs (31.6μM afatinib, 10μM sorafenib or 5.62μM ponatinib) in combination with or without ISRIB (200nM) or salubrinal (20μM) for 24-hr. Data were presented as mean ± SEM (n=3) and analyzed using ANOVA analysis. *p < 0.05, **p < 0.01, ***p < 0.001 versus the DMSO vehicle control group. ^#^p < 0.05, ^##^p < 0.01, ^###^p < 0.001 versus the TKI treatment alone.
