# Supplementary material for: Three tyrosine kinase inhibitors cause cardiotoxicity by inducing endoplasmic reticulum stress and inflammation in cardiomyocytes
Source: BMC Med. 2023 Apr 17;21:147. doi: 10.1186/s12916-023-02838-2 (PMC10108821; doi:10.1186/s12916-023-02838-2)
Supplement: Supplementary file 4 — Additional file 4. Supplementary research methods and details on the reagent or resource used in the experiment. [file 12916_2023_2838_MOESM4_ESM.docx]

**Three tyrosine kinase inhibitors cause cardiotoxicity by inducing endoplasmic reticulum stress and inflammation**

Huan Wang^1,#,*^, Yiming Wang^1,#^, Jiongyuan Li^1^, Ziyi He^1^, Sarah A. Boswell^2^, Mirra Chung^2^, Fuping You^1^, Han Sen^3^

1. Institute of Systems Biomedicine, School of Basic Medical Sciences, Peking University Health Science Center, Beijing, 100191, China

2. Laboratory of Systems Pharmacology, Department of Systems Biology, Harvard Medical School, Boston, Massachusetts 02115, USA

3.Key Laboratory of Carcinogenesis and Translational Research (Ministry of Education), Peking University Cancer Hospital & Institute, Beijing 100142, China.

* Corresponding author, email: [huan_sharon_wang@pku.edu.cn](mailto:huan_sharon_wang@pku.edu.cn)

# these authors contributed equally

**Materials and methods:**

**Animal study**

All experiments were performed in accordance with relevant guidelines and regulations approved by the Institutional Animal Care and Use Committee (IACUC) at Peking University Health Science Center. Sprague-Dawley rats (170-200g) were purchased from Beijing Vital River Laboratory Animal Technology. Forty-eight male rats were randomly divided into six groups, with 8 animals per group. The treatment for each group is as follows: ponatinib (15mg/kg) gavage for 3 days, sorafenib (50mg/kg) for 3 days, 0.5% methycellulose for 3 days, ponatinib (15mg/kg) gavage for 7 days, sorafenib (50mg/kg) for 7 days, 0.5% methycellulose for 7 days, in which 0.5% methycellulose was regarded as control. The gavage was done daily starting at 10am. At the end of the experiments, rats were euthanized by CO2 about 5~6 hours after the last gavage, and then hearts were harvested for further study. Left ventricular muscle was dissected and divided into 3 parts with similar size, one of which was used for RNA extraction and qRT-PCR.

**Immunofluorescence**

Cells cultured in multi-well plates were fixed with 4% paraformaldehyde (Sigma) for 30 minutes at room temperature, washed 3 times with PBS, and permeabilized with 0.2% Tritonx100 (Applygen) for 15 minutes. Then, cells were blocked with 5% normal goat serum (Bioss) for 1-hr at room temperature and probed with primary antibodies against TNNT2 (proteintech) or NF-κB (CST) overnight at 4°C. Secondary antibodies (Jackson Immuno Research Laboratories, Inc.) and 0.4ug/mL DAPI (Coolaber) were applied at room temperature for 1 hour. Immunofluorescence pictures were taken by ImageXpress Micro Confocal-AWES (Molecular Devices).

**Cell titer glo**

ATP content was measured using the CellTiter-Glo Luminescent Cell Viability Assay (Promega) following the manufacturer’s protocol. After adding CellTiter-Glo reagents and incubation, luminescence was quantified using a Synergy H1 plate reader (BioTek Instruments). ATP standards were prepared from 0.01 to 5 μM. For each drug, we fitted a four-parameter log-logistic model to calculate ED50 at day 5 (Fig S1). To rank the cellular toxicity of the eight TKIs (Fig 1T), we calculated $(EC50-Cmax)/(EC50+Cmax)$ and arranged from the smallest to the biggest for increasing cellular toxicity. If C_max_ is bigger than EC50, this drug is likely to be concordantly toxic *in vitro* and *in vivo*; whereas if C_max_ is lower than EC50, the drug might not reach an effective dose to kill cardiomyocytes *in vivo*.

**CardioExcyte® 96 microelectrode array**

HELP hiPSC-CMs were seeded evenly into the 96-well microelectrode array, NSP-96 0.6mm sensor plate (Nanion technologies), at 50,000 cells per well. Measurements were started after 3-5 days of culture of these cells and until the impedance and the extracellular field potential are stable. Measurements were taken a few times before drug addition, and every 30~60 minutes after the drugs were added at 2x concentration. Values were averaged from 3 wells per group. Beat rate, amplitude of impedance, base impedance and FPDc were calculated by the Cardio Excyte Control 96 software package (Nanion Technologies).

**Seahorse assay**

NRCMs were seeded evenly into the Seahorse XF96 cell culture plates (Agilent) and cultured in DMEM high glucose with 2% FBS. About 2 hours before the test, cells were moved to a non-CO_2_ incubator at 37 ° C and incubated for 1 hour. 1 hour prior to the test, culture media was replaced with the Seahorse Basal DMEM at 37°C (with appropriate concentration of glucose, 4mM L-glutamine, 1 mM sodium pyruvate and 1% penicillin-streptomycin solution) and incubated in a non-CO_2_ incubator at 37°C for 1 hour. Drugs were prepared at 8x to 10x concentrations with the corresponding seahorse media as in each well and added into ports of Seahorse XF96 cartridge plates which have been hydrated overnight. XFe96 Extracellular Flux Analyzer (Agilent) was used to measure oxygen consumption rate and extracellular acidification rate of cells with different drug treatments.

**CCK8 assay**

For CCK8 assays, NRCMs were cultured in 384-well plates and drugs were added automatically using HP D300 Digital Dispenser (Tecan). Drug regimen was randomized to minimize bias of well position. After treatment, 10 μL CCK8 detection reagent (TargetMol) was applied per 100µl of culture medium, and cells were incubated in the cell incubator for 2 to 4 hours. Absorbance at 450nm was read with a FlexStation 3 microplate reader.

**ROS and lipid peroxidation staining**

Cells were treated with vehicle control, 10µM afatinib, 10µM sorafenib or 5.62µM ponatinib for 3 and 24 hours in DMEM (Gibco) with 2% FBS (VISTECH). For ROS assessment, cells were washed with PBS once and stained with 10μM H_2_DCFHDA (MCE) in phenol red-free DMEM (Procell) in a humid incubator with 5% CO_2_ at 37°C for 30 minutes. Cells were washed with PBS twice and dissociated to single cells before flow cytometry analysis at the FITC channel. For lipid peroxidation measurement, cells were washed with PBS once and stained with 2μM C11-bodipy^581/591^ (Cayman) in phenol red-free DMEM (Procell) in a humid incubator with 5% CO_2_ at 37°C for 30 minutes. Cells were washed with PBS twice and dissociated to single cells before flow cytometry analysis at both the FITC and the PE channels. Fluorescence of the oxidized C11-bodipy were acquired through the FITC channel (Ex/Em=488/520nm) and that of the non-oxidized C11-bodipy through the PE channel (Ex/Em=555/624nm).

**Calcium staining**

Cells were incubated with the dye working solution, containing 5µM Calbryte™ 520 AM (AAT Bioquest), 0.04% Pluronic® F-127 (Beyotime) and 1 mM Probenecid (AAT Bioquest) in Phenol red-free DMEM (Procell) in a humid incubator with 5% CO_2_ at 37°C for 30 minutes. Cells were washed with PBS once and the media once to remove excess dyes. After staining, cells were treated with vehicle control, 10µM afatinib, 10µM sorafenib or 5.62µM ponatinib in Phenol red-free DMEM (Procell) with 1 mM Probenecid. Images of the calcium dye fluorescence were acquired through the filter Ex/Em=467.5/520nm at 0, 0.5, 1 and 3 hours using ImageXpress Micro Confocal (Molecular Devices). Cells were kept in a humid environment with 5% CO_2_ and 37°C during imaging.

**Propodium Iodide (PI) staining**

Cells were treated with vehicle control, 10µM afatinib, 10µM sorafenib or 5.62µM ponatinib in combination with or without 200nM ISRIB or 10μM 4μ8c for 24-hr. After treatment, cells were stained with 5 μg/mL Propidium iodide (Sigma-Aldrich) mixed with Hoechst33342 (Solarbio) in Phenol red-free DMEM (Procell) in a 5% CO_2_ incubator at 37°C for 30 minutes. Images of PI staining were acquired through the filter Ex/Em=555/624nm and of the nuclei staining through the filter Ex/Em=405/452 using ImageXpress Micro Confocal-AWES (Molecular Devices).

**FDA adverse event reporting system (FAERS) Analysis**

Cardiotoxicity-related adverse drug reactions (ADRs) data for eight tyrosine kinase inhibitors (TKIs) were obtained from the US Food and Drug Administration Adverse Event Reporting System (FAERS) covering the period from July 2014 to December 2020. The demographic, pharmaceutical, and ADR information was extracted from the "demographic file," "drug file," and "reaction file," respectively, and merged using the "primaryid" as the key. Duplicates were removed based on the combination of "primaryid," "caseid," and "fda_dt." In total, 24,666,290 events were detected. To standardize drug names, the active ingredients were extracted from the "prod_ai" field and used to filter the drugs of interest. To minimize the impact of multiple drug interactions on the results, ADRs were included in the analysis only TKIs of interest were considered the primary suspect drug. All ADRs were documented using the Medical Dictionary for Regulatory Activities (MedDRA), and relevant preferred terms (PTs) related to cardiotoxicity were selected based on MedDRA 24.0 (Supplemental Material I). In Supplemental Material I, the PTs were grouped into "Terms" and further classified based on their relevance using "classifications."

The reporting odds ratio (ROR) method was used to calculate the propensity of TKI-induced cardiotoxicity events compared with all other drugs. The RORs were calculated using number $N_{1}$ (events of interest caused by the drug of interest), number $N_{2}$ (any other events caused by the drug of interest), number $N_{3}$ (events of interest caused by any other drugs), number $N_{4}$ (all other events caused by any other drugs) using the following equation:

$$ROR=\frac{N_{1}/N_{3}}{N_{2}/N_{4}}$$

The 95% confidence interval (CI) of ROR was calculated by equation $95\%CI=e^{InROR\pm1.96\sqrt{(\frac{1}{N_{1}}+\frac{1}{N_{2}}+\frac{1}{N_{3}}+\frac{1}{N_{4}})}}$. A significant difference was identified when the lower limit of the 95% confidence interval (CI) of the reporting odds ratio (ROR) was greater than 1. A heatmap was plotted out to illustrate the relationship between the RORs of TKIs and cardiotoxicity-related adverse drug reactions (ADRs) using RStudio version 2021.09.0 and the pheatmap package version 1.0.12. The data was normalized and organized using the Ward's method for clustering. Only the adverse reaction terms that were found to have a significant signal for any TKI were included in the heatmap. The significant signals of RORs were labeled with an asterisk.

**Quantitative Realtime PCR (QPCR)**

RNA was isolated based on a standard protocol using TRIzol reagent (Vazyme). TRIzol (1ml per 1 well of a 6-well plate) was used to lyse cells and chloroform was added for RNA separation. Samples were shaked vigorously by hand for 15 seconds and incubated at room temperature for 2-3 minutes. RNA was present in the aqueous phase after centrifuge at 12000 x g for 15 minutes. Isopropyl alcohol was mixed to precipitate RNA from the aqueous phase. RNA precipitations were washed with 75% ethanol once and re-dissolved in RNase-free water. 1 μg total RNA was used for cDNA synthesis using a reverse transcription kit (Bioman). We then measured expression of genes of interest with SYBRR Green qPCR Mix (Bioman) using Applied Biosystems 7500 Quantitative PCR Instrument. Gene expression was normalized to the 18S ribosomal RNA expression and primers were listed in Primers Table.

**Primers Table**

| Gene Name/Conventional Name | Species | Primer Name | Sequences (From 5’ to 3’) |
| --- | --- | --- | --- |
| Anp | Rattus norvegicus | Forward | ATCTGCCCTCTTGAAAAGCA |
|  |  | Reverse | GGATCTTTTGCGATCTGCTC |
| Atf4 | Rattus norvegicus | Forward | CTTCTCCAGGTGTTCCTCGTT |
|  |  | Reverse | GCTCAGCCCTCTTCTTCTG |
| Atf6 | Rattus norvegicus | Forward | GATTTGATGCCTTGGGAGTC |
|  |  | Reverse | GGACCGAGGAGAAGAGACAG |
| Hspa5/Bip | Rattus norvegicus | Forward | CCCAGGTCAAACACGAGGA |
|  |  | Reverse | GCGGCAAGCAACCAAGGAT |
| Bnp | Rattus norvegicus | Forward | ATCGGCGCAGTCAGTCGCTT |
|  |  | Reverse | GGTGGTCCCAGAGCTGGGGAA |
| Chac1 | Rattus norvegicus | Forward | CCTTCCACAGGGGCAGCGATAAGAT |
|  |  | Reverse | AACCTGGTATGCCACACCCCAAGTG |
| Ddit3/CHOP | Rattus norvegicus | Forward | GGCTTTGGGAGGTGCTTGTG |
|  |  | Reverse | CTGCCTTTCGCCTTTGAGAC |
| Dnajb9 | Rattus norvegicus | Forward | GCCGAACAGGACGAAGGTTG |
|  |  | Reverse | ACTGACTGTGGAGTTGCCAT |
| Il1b/Il-1β | Rattus norvegicus | Forward | CACACACTAGCAGGTCGTCA |
|  |  | Reverse | CCTATGTCTTGCCCGTGGAG |
| Il6/Il-6 | Rattus norvegicus | Forward | AGACTTCCAGCCAGTTGCCT |
|  |  | Reverse | CTGACAGTGCATCATCGCTG |
| Myh6 | Rattus norvegicus | Forward | TGACGTCACCTCCAACATGG |
|  |  | Reverse | AGCTGGGAAATCAGTGCCTC |
| Nfkb1/Nf-κB | Rattus norvegicus | Forward | CCACTGTCAACAGATGGCCC |
|  |  | Reverse | TGTCTGTGAACATCCGTGGG |
| Tnf/Tnf-α | Rattus norvegicus | Forward | CAGATGGGCTGTACCTTATC |
|  |  | Reverse | GGTATGAAATGGCAAATCGG |
| Trib3 | Rattus norvegicus | Forward | AGAGTCCTGGAACGGGTATC |
|  |  | Reverse | AGTTGCGTCGATTTGTCTTC |
| Txnip | Rattus norvegicus | Forward | GTGTCAGTCTCCGCTCGAAT |
|  |  | Reverse | ATAGCTGCTTTGGGGACCAC |
| Xbp1s | Rattus norvegicus | Forward | CTGAGTCCGCAGCAGGTG |
|  |  | Reverse | TTCCAGCTTGGCTGATGAGG |
| 18s rRNA | Rattus norvegicus | Forward | AGTCCCTGCCCTTTGTACACA |
|  |  | Reverse | CGATCCGAGGGCCTCACTA |

**Western blot**

Cells were homogenized in the RIPA lysis buffer (Applygen) containing protease and phosphatase inhibitor cocktails (Bimake). The extract was spun at 12,000xg for 20min at 4°C. Total protein was measured by BCA (Thermofisher) and resolved by sodium dodecyl sulfate–polyacrylamide gel electrophoresis (SDS-PAGE). Proteins were blotted to polyvinylidene difluoride membranes (Amersham). Blots were blocked with non-fat milk (BBI Life Sciences) and incubated with antibodies against ATF6 (proteintech), phospho-eIF2α (ABclonol), XBP1 (ABclonol) and GAPDH (proteintech). Goat anti-rabbit IgG, goat anti-mouse IgG were incubated with the blots for 1 hour at room temperature. After 3 washes with 1xTBST (50 mM Tris-Cl, 150 mM NaCl, 0.1% Tween 20, pH 7.5), the blots were developed with the ECL Plus Kit (Absin). Relative protein expression was quantified by optical density and normalized to GAPDH.

**H9C2 culture and treatment**

H9C2 cells were a gift from Dr. Rong Qi in Peking University Health Science Center (they were originally purchased from the Cell Bank/Stem cell bank, Chinese Academy of Science). Cells were cultured in DMEM (Gibco) containing 10% FBS (VISTECH), and 1% penicillin-streptomycin (Solarbio) in a humid incubator with 5% CO_2_ at 37°C. Media was changed to DMEM (Gibco) containing 2% FBS 12 hours before treatment. All treatments were performed under DMEM containing 2% FBS.

**Key Resource Table**

| **REAGENT or RESOURCE** | **SOURCE** | **IDENTIFIER** |
| --- | --- | --- |
| **Chemicals** | | |
| Magnesium chloride | Invitrogen | AM9530G |
| Paraformaldehyde | Sigma | P6148-500g |
| 4μ8c | selleck | S7272 |
| 5-bromo-2-deoxyuridine | Shanghai Yuanye | S18116 |
| Afatinib | MedChemExpress (MCE) | HY-10261/CS-0043 |
| AMPure XP | Beckman | A63880 |
| C11-bodipy 581/591 | Cayman | 27086 |
| Calbryte™ 520 AM | AAT Bioquest | 20650 |
| Chloroform | Tongguang | 12322 |
| Phosphatase inhibitor cocktail | Bimake | B15001 |
| Crizotinib | MCE | HT-50878/CS-0029 |
| DAPI | Coolaber | CD4261 |
| Dasatinib | MCE | HY-10181/CS-0100 |
| Dimethyl sulfoxide (DMSO) | Sigma-Aldrich | D8418-100ML |
| ECL Plus Kit | Absin | abs920 |
| Ethanol | Tongguang | 12404 |
| Gefitinib | MCE | HY-50895/CS-0124 |
| H_2_DCFDA | MCE | HY-D0940 |
| Hank's Balanced Salt Solution (HBSS) | Gibco | C14175500BT |
| Hoechst33342 | Solarbio | C0030 |
| Isopropyl alcohol | Tongguang | 12409 |
| ISRIB | Sellleck | S0706 |
| Sodium hydroxide (NaOH) | Sigma | S5881 |
| Nilotinib | MCE | HY-10159/CS-0102 |
| Nuclease-free water | Invitrogen | AM9930 |
| Pluronic® F-127 | Beyotime | ST501 |
| Protease Inhibitor Cocktail (EDTA-Free, 100X in DMSO) | Bimake | 14001 |
| Ponatinib | MCE | HY-12047/CS-0204 |
| Probenecid | AAT Bioquest | 20060 |
| Propidium iodide | Sigma-Aldrich | P4107 |
| RIPA lysis buffer | Applygen | C1053 |
| Salubrinal | Selleck | S2923 |
| Sodium chloride | Tongguang | 112007 |
| Sorafenib, free base | LC Laboratories | S-8599 |
| Sunitinib | MCE | HY-10255A/CS-1670 |
| Tris | Genview | 77-86-1 |
| TRIzol | Vazyme | R401-01 |
| Tritonx100 | Applygen | A1009 |
| Tween 20 | Dingguo, Beijing | DH358-4 |
| **Biological Reagents** | | |
| Alexa Fluor® 488-conjugated AffiniPure Goat Anti-Rabbit IgG (H+L) | Jackson Immuno Research Laboratories | 111-545-003 |
| ATF6 Rabbit polyclonal antibody | Proteintech | 24169-1-AP |
| Bovine serum albumin (BSA) | BBI Life Sciences | A1087 |
| Cardiac Troponin T Polyclonal antibody | Proteintech | 26592-1-AP |
| Collagenase | Gibco | 17101-105 |
| Dulbecco's Modified Eagle Medium (DMEM, high glucose) | Gibco | 11966-025 |
| DMEM (high glucose, without phenol red, with HEPES) | Procell | PM150226 |
| DNA Clean & Concentrator-5 | ZYMO | D4013 |
| Fetal Bovine Serum (FBS) | VISTECH | SE100-011 |
| GAPDH Monoclonal antibody | Proteintech | 60004-1-Ig |
| Goat serum | Bioss | C0005 |
| Maxima H Minus cDNA Synthesis Master Mix, with dsDNase | Thermo | M1682 |
| Maxima H Minus Reverse Transcriptase | Thermo | EP0752 |
| NF-κB p65 (D14E12) XP® Rabbit mAb | Cell Signaling Technology (CST) | 8242 |
| Non-fat milk | BBI Life Sciences | NB0669 |
| Penicillin-streptomycin solution (100X) | Solarbio | P1400 |
| Phospho-eIF2a-S51 Rabbit mAb | Abclonol | AP0692 |
| RNASEOUT | Invitrogen | 10777019 |
| Thermolabile Exonuclease I | NEB | M0568S |
| Trypsin | Macklin | T6325 |
| Trypsin-EDTA solution,0.25% (without phenol red) | Solarbio | T1300 |
| XBP1 rabbit pAb | Abclonol | A1731 |
| **Critical Commercial Assays** | | |
| Advantage 2 PCR Kit | Takara | 639207 |
| BCA | Thermo | TL276913 |
| Cell Counting Kit-8 (CCK-8) | TargetMol | C0005 |
| RTIII All-in-One Mix with dsDNase | Bioman | BIO9293 |
| SYBRR Green qPCR Mix | Bioman | BIO9508 |
| CellTiter-Glo Luminescent Cell Viability Assay | Promega | G7570 |
| **Deposited Data** | | |
| Images will be deposited | <https://idr.openmicroscopy.org/cell/> |  |
| Transcriptome data will be deposited | Gene Expression Omnibus |  |
| **Experimental Models: Cell Lines** | | |
| H9C2 | Cell Bank/Stem cell bank, Chinese Academy of Science | GNR-5 |
| Neonatal Rat Cardiac Myocytes (NRCM) from Sprague-Dawley rats | Beijing Vital River Laboratory Animal Technology |  |
| **Software and Algorithms** | | |
| Fiji | https://imagej.net/software/fiji/ |  |
| Graphpad Prism | https://www.graphpad.com/ |  |
| Adobe illustrator | https://www.adobe.com/products/illustrator.html |  |
| **Other** | | |
| 6-well cell culture plate | Sorfa | 220100 |
| 96-well glass bottom plate | Cellvis | P96-1.5H-N |
| 96-well cell culture plate | Sorfa | 220400 |
| 384-well cell culture plate | Corning | 353961 |
| Magnetic stand-96 | Invitrogen | AM10027 |
