# Supplementary material for: Three tyrosine kinase inhibitors cause cardiotoxicity by inducing endoplasmic reticulum stress and inflammation in cardiomyocytes
Source: BMC Med. 2023 Apr 17;21:147. doi: 10.1186/s12916-023-02838-2 (PMC10108821; doi:10.1186/s12916-023-02838-2)

p-eIF2α (afatinib, sorafenib and ponatinib)


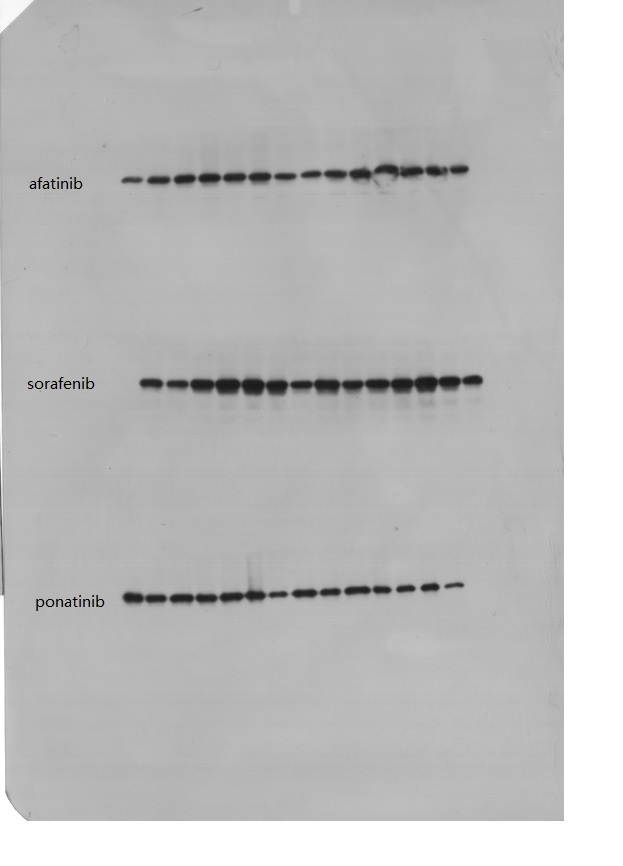


.

XBP1s (afatinib, sorafenib and ponatinib) in two repeats


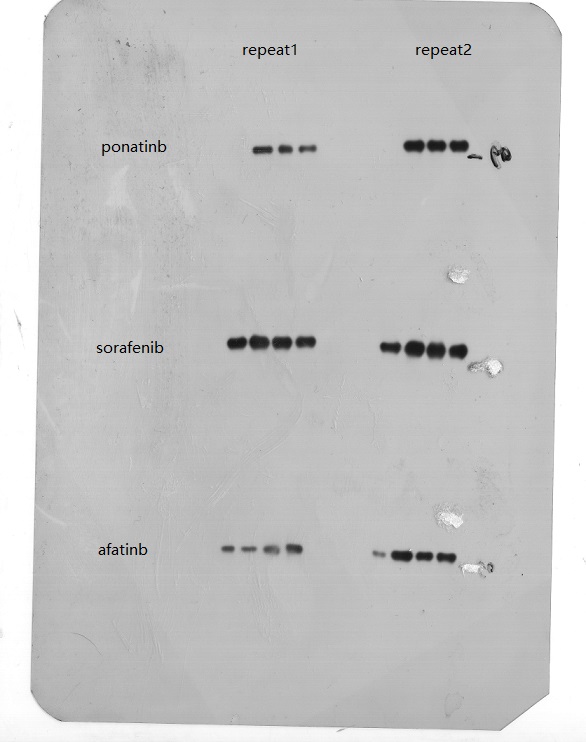


ATF6 and GAPDH (afatinib and ponatinib)


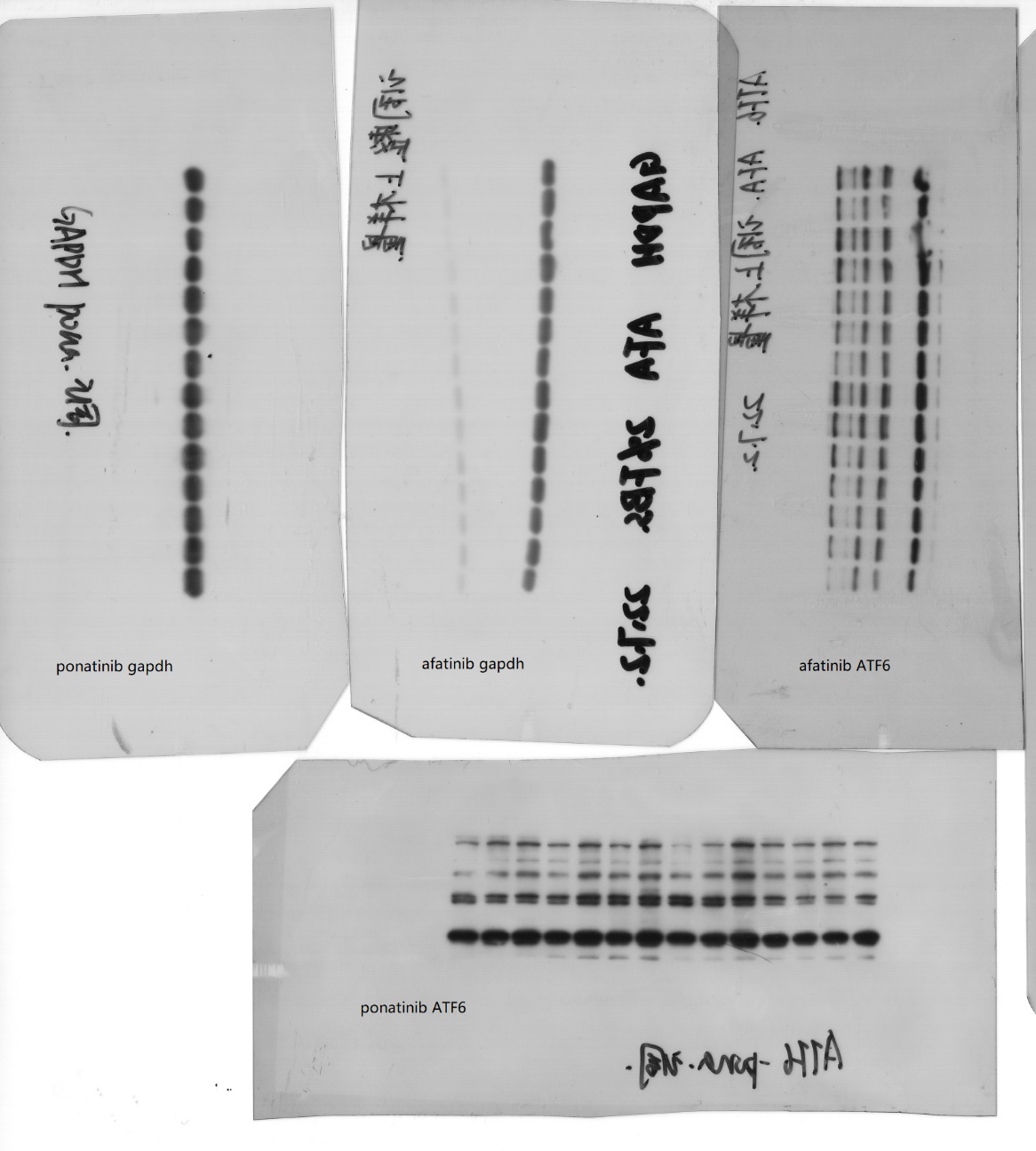


ATF6 and GAPDH (sorafenib)


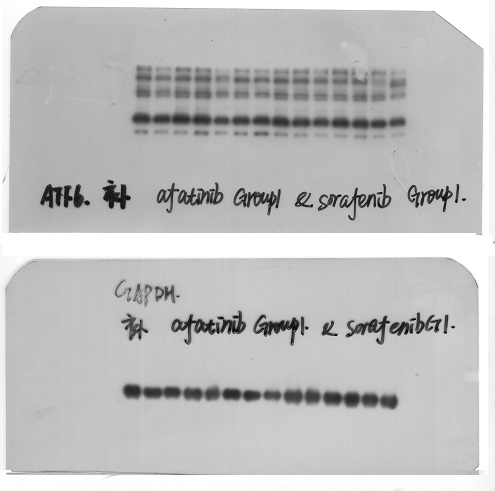


ATF6 (ponatinib)


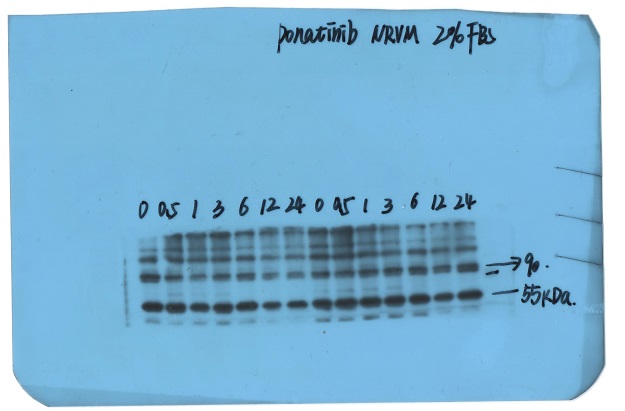


ATF6 (sorafenib)


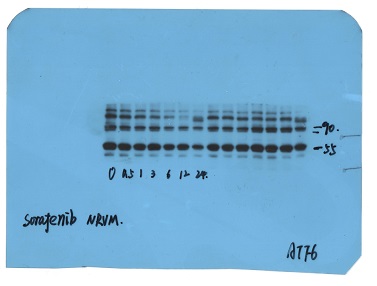


GAPDH (sorafenib and ponatinib)


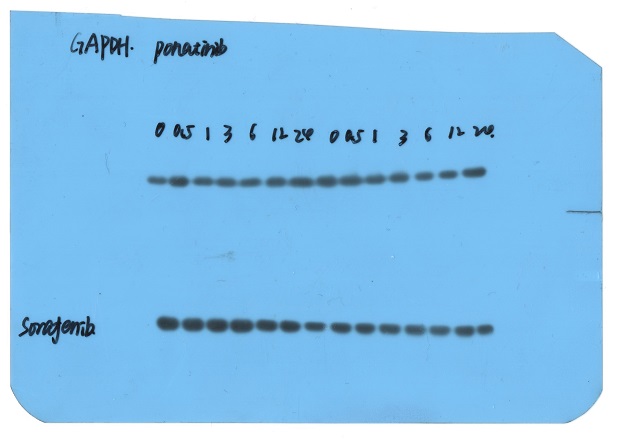

Supplement: Supplementary file 5 — Additional file 5. Original western blot gel images, related to Fig. 4. [file 12916_2023_2838_MOESM5_ESM.docx]
